# Adhesive Functions or Pseudogenization of Type Va Autotransporters in *Brucella* Species

**DOI:** 10.3389/fcimb.2021.607610

**Published:** 2021-04-27

**Authors:** Magalí G. Bialer, Mariana C. Ferrero, M. Victoria Delpino, Verónica Ruiz-Ranwez, Diana M. Posadas, Pablo C. Baldi, Angeles Zorreguieta

**Affiliations:** ^1^ Fundación Instituto Leloir (FIL), IIBBA (CONICET-FIL), Buenos Aires, Argentina; ^2^ Facultad de Farmacia y Bioquímica, Cátedra de Inmunología, Universidad de Buenos Aires, Buenos Aires, Argentina; ^3^ Instituto de Estudios de la Inmunidad Humoral (IDEHU), CONICET-Universidad de Buenos Aires, Buenos Aires, Argentina; ^4^ Instituto de Inmunología, Genética y Metabolismo (INIGEM), CONICET-Universidad de Buenos Aires, Buenos Aires, Argentina; ^5^ Departamento de Química Biológica, Facultad de Ciencias Exactas y Naturales, Universidad de Buenos Aires, Buenos Aires, Argentina

**Keywords:** type Va autotransporter, adhesin, pseudogene, adhesion, Brucella, outer membrane protein (OMP), polar localization, extracellular matrix (ECM)

## Abstract

Adhesion to host cells is a key step for successful infection of many bacterial pathogens and may define tropism to different host tissues. To do so, bacteria display adhesins on their surfaces. *Brucella* is an intracellular pathogen capable of proliferating in a wide variety of cell types. It has been described that BmaC, a large protein that belongs to the classical (type Va) autotransporter family, is required for efficient adhesion of *Brucella suis* strain 1330 to epithelial cells and fibronectin. Here we show that *B. suis* 1330 harbors two other type Va autotransporters (BmaA and BmaB), which, although much smaller, share significant sequence similarities with BmaC and contain the essential domains to mediate proper protein translocation to the bacterial surface. Gain and loss of function studies indicated that BmaA, BmaB, and BmaC contribute, to a greater or lesser degree, to adhesion of *B. suis* 1330 to different cells such as synovial fibroblasts, osteoblasts, trophoblasts, and polarized epithelial cells as well as to extracellular matrix components. It was previously shown that BmaC localizes to a single bacterial pole. Interestingly, we observed here that, similar to BmaC, the BmaB adhesin is localized mostly at a single cell pole, reinforcing the hypothesis that *Brucella* displays an adhesive pole. Although *Brucella* species have strikingly similar genomes, they clearly differ in their host preferences. Mainly, the differences identified between species appear to be at loci encoding surface proteins. A careful *in silico* analysis of the putative type Va autotransporter orthologues from several *Brucella* strains showed that the *bmaB* locus from *Brucella abortus* and both, the *bmaA* and *bmaC* loci from *Brucella melitensis* are pseudogenes in all strains analyzed. Results reported here evidence that all three autotransporters play a role in the adhesion properties of *B. suis* 1330. However, *Brucella* spp. exhibit extensive variations in the repertoire of functional adhesins of the classical autotransporter family that can be displayed on the bacterial surface, making them an interesting target for future studies on host preference and tropism.

## Introduction

*Brucella* species belong to the *Alphaproteobacteria* group and are responsible for a worldwide spread zoonosis called brucellosis. There are twelve species of *Brucella* identified so far, each one with particular host preferences. The most relevant species regarding their negative impact on the livestock industry and human health are *Brucella abortus* (that infects cattle), *Brucella melitensis* (goats), and *Brucella suis* (swine). Human brucellosis is mostly caused by accidental contamination with reproductive or mammary secretions from infected animals and is characterized by a flu-like fever which, if untreated, may progress to a chronic stage of the disease and the appearance of serious complications such as arthritis, endocarditis, osteoarthritis, spondylitis, hepatosplenomegaly, and neurological disorders (Pappas et al., 2006; Buzgan et al., 2010; Giambartolomei and Delpino, 2019).

The classification of *Brucella* species is controversial because the similarity of their genomes is higher than 95% (Foster et al., 2009; Whatmore, 2009). However, it is clear that each species exhibits affinity for a particular host (or a few hosts). The genetic and molecular bases of host preferences for different *Brucella* species are still unknown. It is interesting that several of the few genomic differences between species correspond to genes associated with surface proteins, suggesting that these factors might have a role in host specificity (Paulsen et al., 2002; Halling et al., 2005).

*Brucella* is an intracellular pathogen that can replicate and establish an infection niche in multiple cell types, such as macrophages, epithelial cells, synoviocytes, osteoblasts, fibroblasts, hepatocytes, dendritic cells, and trophoblasts (Billard et al., 2005; Delpino et al., 2009; Ferrero et al., 2009; Delpino et al., 2010; von Bargen et al., 2012). It has been shown that initial adhesion to host cells and to components of the extracellular matrix (ECM) is crucial for a successful infection by intracellular pathogens. Bacterial proteins called adhesins usually mediate the binding process to host cell receptors or ligands and ECM components, avoiding clearance of the bacteria by fluids of the host. In the case of intracellular pathogens, adhesins also promote invasion of host cells (Pizarro-Cerda and Cossart, 2006; Patel et al., 2017). It was previously proposed that *Brucella* is capable to bind sulfated and sialic acid residues and ECM components such as fibronectin, collagen, hyaluronic acid, and vitronectin (Campbell et al., 1994; Castaneda-Roldan et al., 2004; Posadas et al., 2012; Ruiz-Ranwez et al., 2013a; Ruiz-Ranwez et al., 2013b; ElTahir et al., 2019). By phage display screening a large 340 kDa fibronectin-binding protein of *B. suis* strain 1330 (BmaC), belonging to the Type Va secretion system family, was identified. It was shown that BmaC contributes to *Brucella* adhesion to epithelial cells and localizes to the new bacterial pole generated after asymmetrical division (Posadas et al., 2012; Ruiz-Ranwez et al., 2013a; Ruiz-Ranwez et al., 2013b).

The type V secretion systems, also referred as autotransporters (subfamilies Va–Ve), play important roles in the interaction of several pathogens with their hosts (Meuskens et al., 2019). While in the subclass Va (also called classical autotransporters) the passenger and secretion domains (see below) are integrated into the same protein, the type Vb consists in two distinct polypeptides encoded by different genes. The type Vc has a similar topology to the type Va but forms a trimeric structure. The type Vd is a hybrid between type Va and type Vb, and in the type Ve autotransporters the order of the domains is inverted compared to that of type Va.

Proteins from the type Va subclass contain a signal peptide at the N-terminal end that is recognized by the Sec machinery, which mediates protein translocation through the inner membrane and into the periplasmic space. Once there, the β-barrel domain encoded by the C-terminal end is inserted into the outer membrane forming a pore through which the passenger (and functional) domain is translocated to the extracellular medium (Benz and Schmidt, 2011). Analysis of the barrel domains shows at least 14 types of β-barrel structures, according to differences in the arrangements of conserved motifs that define the type Va autotransporter subfamily in general (Celik et al., 2012). Several evidences indicate that the term “auto” does not represent the secretion mechanism of autotransporters since other proteins are required for their proper secretion. The β-barrel assembly machinery (BAM) was found to be required for translocation of several autotransporters (Leyton et al., 2012). Furthermore, evidence was presented indicating that a new system called Translocation and Assembly Module (TAM) was also necessary for the efficient translocation of autotransporters, including those of *Brucella* (Selkrig et al., 2012; Bialer et al., 2019).

*Brucella suis* 1330 genome encodes other surface factors required for efficient adhesion to epithelial cells and components of the ECM. We have previously shown that BtaE and BtaF are crucial for bacterial pathogenicity (Ruiz-Ranwez et al., 2013a; Ruiz-Ranwez et al., 2013b). Although BtaE and BtaF proteins were detected in a reduced number of bacterial cells during *in vitro* growth, similar to BmaC, they also showed polar localization (Ruiz-Ranwez et al., 2013a; Ruiz-Ranwez et al., 2013b). Interestingly, the BtaE trimeric autotransporter of *B. suis* 1330 and *B. abortus* 2308 showed some differences in size, number of repetitive motifs and regulatory mechanisms (Sieira et al., 2017).

In addition to BmaC, *B. suis* 1330 encodes two other type Va autotransporters, named as BmaA and BmaB. It was reported that a mutant of *B. suis* 1330, deficient in BmaB (previously called OmaA), is cleared from spleens of BALB/c mice faster than the wild type strain (between the third and the ninth week post infection), suggesting that BmaB is required during the chronic phase of infection (Bandara et al., 2005).

A preliminary genome analysis of the *bmaA* and *bmaB* orthologues from the *B. melitensis* 16M and *B. abortus* 2308 reference strains showed that they correspond to pseudogenes. Thus, the question arises of whether pseudogenization on these loci is a generalized event in the genomes of most strains of these *Brucella* species and if the BmaA and BmaB proteins encoded by the *B. suis* 1330 genome fulfill some adhesive function. To explore this, the contribution of Bma proteins of *B. suis* 1330 to efficient adhesion to different cell types in culture was analyzed. In addition, we performed a careful *in silico* analysis of the *bma* orthologues (including *bmaC*) from numerous strains of *B. suis*, *B*. *abortus* and *B. melitensis.* Our findings open interesting hypotheses regarding the contribution of Bma proteins to host preferences.

## Materials and Methods

### Bacterial Strains, Cell Culture, and Media

*Escherichia coli* strains used in this study (Max Efficiency™ DH5α, Thermo Fisher Scientific, Waltham, MA, USA) were grown at 37°C in lysogeny broth (LB) medium. *Brucella suis* M1330 (ATCC 23444) and derivative strains were grown at 37°C in Bacto tryptic soy broth (TSB; Bacto). When necessary, antibiotics were added: chloramphenicol at 6 µg/ml, kanamycin (Km) at 50 µg/ml, and nalidixic acid (Nal) at 10 µg/ml. All *Brucella* strains used in this study were handled in biosafety laboratories (BSL3) at Leloir Institute (IIBBA-FIL) and Instituto de Investigaciones Biomédicas en Retrovirus y SIDA (INBIRS). Eukaryotic cell lines were cultured in Dulbecco’s modified Eagle’s medium (DMEM; Gibco) supplemented with 5% fetal calf serum (Natocor), at 37°C in a 5% CO_2_ atmosphere.

### Molecular Techniques

All DNA manipulations were carried out by using standard procedures. Sequence data of *B. suis* were obtained from the NCBI Database. The primers used are shown in **Table S1**.

To construct the *bmaA*::stops and the the *bmaB*::Km mutant, a 972 and 1,468 pb amplicon were obtained respectively, using BRA0173F and BRA0173R primers. The amplicons were cloned into a pGemTeasy vector. BamHI restriction enzyme was used to add the Stops region from the Omega Cassette (Prentki & Krisch, 1984) in the middle of *bmaA* amplicon. SmaI restriction enzyme was used to introduce the Kanamycin Cassette (Quandt and Hynes, 1993) in the middle of *bmaB* amplicon. The plasmids were electroporated into *B. suis* wild type strain and double recombinant clones were selected. The interruption of the open reading frame (ORF) was confirmed by PCR using Omega_stopsR primer and nptIIR primer, respectively.

To provide a phenotypic confirmation of the mutants, the entire *bmaA or bmaB* gene (including its own promoter) was cloned into a broad-host-range plasmid. Amplicons of 3,519 and 4,905 bp were amplified respectively using the following primers: BR0173_Fw_Prom_XhoI, BRA0173_Bam_Rev,BR2013_Fw_Prom_Fwd and BR2013_Sac_rev. The amplified product was cloned into the pBBR1MCS-1 mid-copy-number plasmid (Kovach et al., 1995), obtaining the pBBR*bmaA* and pBBR*bmaB* plasmids, which were conjugated into the *B. suis bmaA::*stops or *bmaB*::Km strains. Complemented clones were selected (Cm^R^) and confirmed by PCR.

Construction of the bmaC mutant and the complemented knock-in (KI) strain was already described in a previous study (Posadas et al., 2012).

### Pseudogene Determination

A search was made for sequence similarity using BlastP looking for ORFs that conserve the same position in the genome (synteny). Domain search was performed using Pfam (Finn et al., 2014). Signal peptide sequence and subcellular localization were predicted using SignalP Server (5.0 version) (Almagro Armenteros et al., 2019). The alignments were performed with ClustalOmega (EMBL-EBI) (Madeira et al., 2019). Translation of nucleotide sequences was performed by ExPASy Translate Tool (Artimo et al., 2012). The cases in which both, the signal peptide and the β-barrel domain were found in the same peptide were considered complete and “functional” proteins, and those cases in which the loci were split into two or more ORFs (none containing the signal peptide and the β-barrel simultaneously) were considered pseudogenes.

### Phylogenetic Analysis

Multiple alignment was performed by Muscle. Phylogeny was inferred using the Neighbor-Joining method (Saitou and Nei, 1987). The evolutionary distances were computed using the Poisson correction method (Zuckerkandl and Pauling, 1965) and are expressed as the number of amino acid substitutions per site. Evolutionary analyses were conducted in MEGA X (Kumar et al., 2018).

### Cell Infection Assays

Primary synovial fibroblasts (synoviocytes) were isolated from synovial tissues obtained from patients undergoing total knee replacement surgery in accordance with the guidelines of the Ethical Committee of the INIGEM Institute, as previously described (Scian et al., 2013). Primary osteoblasts were isolated from newborn-mouse calvaria using the method described by Wong and Cohn (Wong and Cohn, 1975). The Swan71 cell line (kindly provided by Dr. Gil Mor, Yale University, New Haven, USA) was obtained by telomerase-mediated transformation of a 7-week cytotrophoblast isolate. Primary cells and the cell lines Caco-2 (ATCC HTB-37™), HT-29 (ATCC HTB-38), Swan71, SW982 (ATCC HTB-93™), and hFOB (ATCC CRL-11372™) were seeded in 24-well plates (5 × 10^4^ cells per well) and inoculated with the different strains (multiplicity of infection [MOI], 100:1). Plates were centrifuged for 10 min at 170 RCF and then incubated in a 5% CO_2_ atmosphere at 37°C for 1 h. To determine the total number of bacteria associated to the cells, wells were washed three times with PBS and treated for 10 min at 37°C with 0.1% Triton X-100 (in PBS). Serial dilutions of the obtained lysates were done in PBS and plated on TSA with the appropriate antibiotics, and CFU were determined. All the results were expressed in reference to the wild-type strain, defined as 100%.

### Binding to ECM Components

Analysis of bacterial binding to extracellular matrix (ECM) components was performed as described elsewhere (Serruto et al., 2009). Briefly, 96-well plates (Nunc Maxisorp) were coated overnight at 4°C with 50 µl of 100 µg/ml solutions of the ligands (Fibronectin, Hyaluronic acid, Type I Collagen), dissolved in phosphate-buffered saline (PBS). Bacteria were grown overnight, washed, and resuspended in PBS to a final concentration of 1 × 10^9^ CFU/ml (optical density OD 1 for *E. coli* and OD 0.2 for *Brucella*). Wells were washed three times with 100 µl of PBS to eliminate unbound ligand. Then, 50 µl of bacterial suspensions were added to each well and incubated at 37°C for 3 h. After incubation, wells were washed three times with PBS to remove non-adherent bacteria, and then adherent bacteria were harvested with trypsin-EDTA (0.05% trypsin [Gibco]–0.5% EDTA [USB]). After incubation for 10 min at 37°C, serial dilutions were done, followed by plating on LB or TSB agar with the appropriate antibiotic, and CFU were determined.

### Biofilm and Autoagglutination Assays

Bacterial attachment to wells of polystyrene (PE) plates was assayed based on a previous protocol (O’Toole et al., 1999). Briefly, starter cultures were grown in appropriate media overnight and diluted on fresh media (1:1,000); then 150 µl of these diluted cultures were seeded in the wells of PE 96-well flat bottom culture plates (Cellstar, Greiner Bio One). These plates were then cultured at 37°C for 24 h at 200 rpm. Unbound bacteria were removed by gently washing the wells three times with NaCl 0.9%, and attached bacteria were quantified by staining with 0.1% (w/v in water) crystal violet (CV) (Acros Organics, Geel, Belgium). Optical density was measured at λ = 595 nm in a Microplate Reader (DTX 880 multimode detector, Beckman Coulter).

To evaluate cell-cell agglutination, starter cultures (see above) were diluted 1:100 and grown for 16 h in an appropriate medium at 37°C with agitation at 200 rpm. The cultures were allowed to settle at room temperature. Bacterial sedimentation was measured comparing the optical density (OD_600_)in the upper portion of the tubes at different time points for a period of 96 h.

### Immunofluorescence Assay

To detect the presence of adhesins on the bacterial surface by immunofluorescence (IF), *B.* suis + pBBR3xFLAG-*bmaB* strain (Bialer et al., 2019) was conjugated with plasmids carrying pole markers pMR10-*aidB-ypf* (Dotreppe et al., 2011) or pMR10-*pdhS-egpf* (Hallez et al., 2007) and transconjugants were selected by Nal, Km, and Cm resistance. For *E. coli* IF, the CC118 + pBBR3xFLAG-*bmaB* strain was used.

Bacteria were cultured in TSB or LB media, and corresponding antibiotics, until OD_600_ 0.6. Then, the cells were harvested by centrifugation (5 min at 5,000 rpm) and washed with PBS.

When required, cells were fixed with 3.7% paraformaldehyde for 15 min at 37°C with gentle shaking. Then the pellet was washed two times with PBS, and once with PBS-1% bovine serum albumin (BSA) and incubated for 40 min. The bacteria were then resuspended in 40 µl of the primary antibody (anti FLAG, SIGMA) diluted 1:500 in PBS-BSA 0.5% and incubated for 40 min at room temperature, with gentle shaking. Bacteria were washed three times with 1% PBS-BSA and then resuspended in 40 µl of Cy3-conjugated anti-mouse antibody (Jackson Immuno Research) diluted 1:250 in PBS-BSA 0.5%, and incubated for 40 min, at room temperature. Bacteria were washed two times with PBS-BSA 1% and once with PBS and resuspended in one volume of PBS. An aliquot of the suspension (3 µl) was mounted on a 1% agarose pad. Samples were covered with a coverslip and viewed under a confocal microscope (Zeiss LSM 880 with Airyscan). The images were analyzed with the ZEN 2.3 software. In all cases, the corresponding viability controls were performed, in liquid and solid medium, after fixation.

### Quantitative Real Time-Polymerase Chain Reaction (RT-qPCR)

For RNA extraction, bacteria were cultured in rich media up to an exponential growth phase. Total RNA was extracted using Epicentre ^®^ Master Pure™ Kit (Illumina). One microgram of RNA was treated with TURBO DNase (Thermo Fisher Scientific, Waltham, MA, USA) and subjected to retro-transcription with Super Script III Reverse Transcriptase and random hexadeoxynucleotides (Thermo Fisher Scientific, Waltham, MA, USA) according to manufacturer’s instructions. cDNAs were then amplified with FastStart Universal SYBR Green Master (Roche, Basel, Switzerland) using the Mx3000P Real Time PCR System cycler (Agilent Technologies, Santa Clara, CA, USA). The results of each target were normalized to *B*. *suis* Translation Initiation Factor-1 (*if-1*, BR0249) mRNA (Eskra et al., 2001). The relative expression levels of the target genes were calculated according to the comparative critical threshold (ΔΔC_T_) method (Livak and Schmittgen, 2001). Three biological samples were measured for each strain. Oligonucleotides used are listed in **Table S1**.

### Statistical Analysis

All values are expressed as mean ± standard deviation. All experiments were performed at least three times in triplicate unless otherwise indicated. Statistical analyses were done using with the GraphPad Prism software. Non-parametric one-way ANOVA test followed by Tukey *post hoc* test was performed. Differences between values were considered significant at *P* < 0.05 (*, *P* < 0.05; **, *P* < 0.001; ***, *P* < 0.0001).

## Results

### BmaA and BmaB From *B. suis* 1330 Are Monomeric Type Va Autotransporters

**Figure 1:**
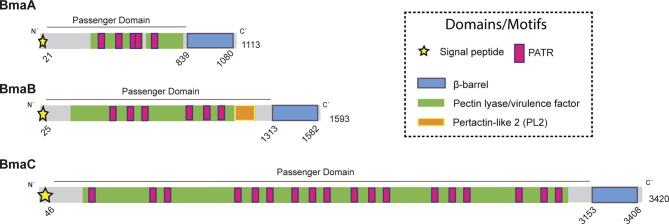
Domain organization of Bma proteins. Schematic representation of BmaA, BmaB, and BmaC, showing functional and structural domains predicted by bioinformatics (SignalP 5.0, Pfam, BLAST). Numbers indicate amino acid positions of the predicted signal peptide, β-barrel domain, and total length of the protein. Modified from Bialer et al., 2020.

BmaA (BRA0173) is encoded on chromosome II, the same as the large BmaC adhesin, and annotated as an outer membrane autotransporter protein of 1,113 amino acids (110 kDa) (Paulsen et al., 2002). Analysis of BmaA using SignalP (5.0 version) predicted a signal peptide with a cleavage site between positions 20 and 21. This signal peptide would be substrate of the SPaseI from the Sec machinery (0.559 likelihood) (Almagro Armenteros et al., 2019). The annotated open reading frame (ORF) of BmaA starts with a GTG codon. It has been reported that the GTG (Val) and TTG (Leu) codons, and to a minor extent CTG (Val), are usually recognized as the initiation site for gene translation in prokaryotes, as alternative codons to the canonical ATG (Met) (Bachvarov et al., 2008). BmaA shows the characteristic C-terminal translocator domain of classical autotransporters, consisting of 12 antiparallel β strands (Jpred4). In the passenger domain, according to the Pfam, HMMER and InterProScan databases, five repetitions associated with autotransporters (PATR) (Doyle et al., 2015) and a pectin lyase-like domain were identified (**Figure 1**). The denomination of this last domain is associated with the function assigned in its original description in plant pathogens but it is also present in virulence factors of mammalian pathogens such as the *Salmonella* P22 phage tailspike protein, and the *Bordetella pertussis* pertactin protein (P.69) (Emsley et al., 1996). According to the NCBI database, the PATR motifs and the pectin lyase-like domain are included in a broader domain of the AIDA-I superfamily (e-value 2.9 e^-30^) (Benz and Schmidt, 1992) (**Figure 1**).

The BmaB protein (BR2013) is a 1593-amino acid protein encoded on chromosome I, which contains all the typical domains of classical autotransporters. An N-terminal signal peptide of 25 residues was predicted by SignalP 5.0 analysis (0.9327 of likelihood) and a C-terminal β-barrel translocator domain predicted by Pfam (**Figure 1**). Similar to BmaA, the passenger domain contains 6 PATR repetitions. In addition, a region identified in Pfam as “pertactin” (PF03212.14) and according to NCBI as “pertactin-like” (PL2 cd01344) (Emsley et al., 1996) was found upstream the translocator domain. However, a functional or structural role of this region cannot be proposed without further studies (Drobnak et al., 2015).

BmaA, B, and C of *B. suis* 1330 show similar overall protein structures, sharing similar motifs and domains. By local alignments (BLAST) BmaA exhibits 46% sequence identity with BmaB (97% of coverage, e-value <1e^−100^) and 41% of identity with BmaC (80% of coverage, e-value <1e^−100^). BmaB shares 41.06% of identity with BmaC (69% of coverage, e-value <1e^−100^). These e-values represent with high confidence that the pairwise alignment is a result of homologous relationships, thus indicating that the three of them are homologous. Phylogenetic analysis of the β-barrel region of Bma proteins and several autotransporters belonging to each barrel-domain “type” (Celik et al., 2012) suggests that the Bma β-barrel regions are related to the BapA-type. In addition, BmaA and BmaB are more closely related between them than with BmaC (**Figure S1**).

### Contribution of Type Va Autotransporters to the Adhesion of *B. suis* 1330 to Different Cell Types and ECM Components

As described above, analysis of BmaA and BmaB proteins from *B. suis* strain 1330 indicates that they could be functional, meaning that both proteins harbor all the information to guarantee their correct translocation through both the inner and the outer membranes. To confirm this hypothesis, the effect of *bmaA* and *bmaB* inactivation in the adhesion of *B. suis* 1330 to different cell types was analyzed. Previous results from our laboratory indicated that BmaC is required for efficient adhesion of *B. suis* 1330 to epithelial cell lines, such as HeLa and A549 (Posadas et al., 2012). Since the contribution of BmaC to the interaction of *B. suis* with other cell types has not been evaluated, the *bmaC* mutant was also analyzed in the present study.

**Figure 2:**
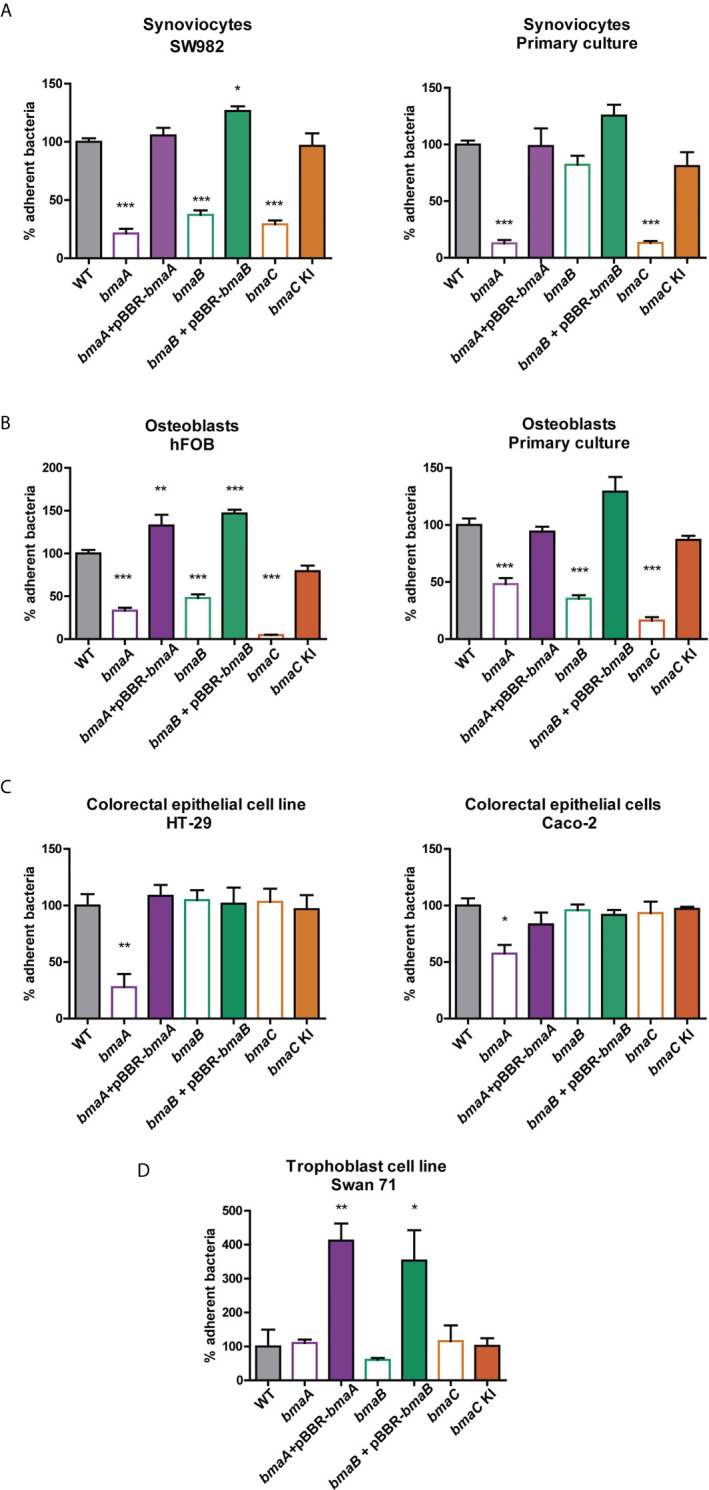
Adherence of *Brucella* strains to hosts cells. **(A)** Synoviocyte cell line (SW982) and primary culture, **(B)** Osteoblast cell line (hFOB) and primary culture, **(C)** colorectal epithelial cell lines (HT-29 and Caco-2) and **(D)** trophoblast cell line (Swan71) were infected with *B. suis* wild type, *bmaA*::*stops* (*bmaA*), *bmaB*::Km (*bmaB*), Δ*bmaC* (*bmaC*) and the respective complemented strains (*bmaA::*st + pBBR*bmaA, bmaB*::Km + pBBR*bmaB*, and *bmaC knock-in –*KI-) using a MOI of 1:100. The percentage of adhered bacteria was determined based on the total of bacteria inoculated per well. The percentage of attached bacteria was expressed relative to the control strain, to which the value of 100% was assigned. The results shown correspond to the mean ± standard deviation (SD) of the average of three tests performed in triplicate. The data were analyzed with ANOVA followed by the Tukey´s *post hoc* test. *, significantly different from control p < 0.05, **p < 0.005, ***p < 0.0001.

As mentioned above, *Brucella* has the ability to infect various cell types including trophoblasts, fibroblasts, and epithelial cells (Billard et al., 2005; Delpino et al., 2009; Ferrero et al., 2009; Delpino et al., 2010; Martirosyan et al., 2011; Scian et al., 2011; von Bargen et al., 2012; Fernandez et al., 2016). In addition, one of the most frequent clinical manifestations of the infection is osteoarticular brucellosis. Furthermore, the adhesion capacity of *Brucella* spp. to osteoblasts (hFOB cell line and primary cultures from newborn mouse calvaria) and synoviocytes (SW982 cell line and primary culture from human synovial tissue) was reported (Delpino et al., 2009; Scian et al., 2012; Salcedo et al., 2013; Scian et al., 2013). Therefore, the role of Bma proteins in the adhesion of *B. suis* 1330 to these cells was evaluated. Interestingly, the three mutants analyzed, *bmaA*::st, *bmaB::*Km, and Δ*bmaC*, showed a decrease in the adhesion to synoviocytes and osteoblasts, both in primary cultures and cell lines (**Figures 2A, B**), with the exception of *bmaB::*Km in primary culture of synoviocytes. The complemented mutants showed adhesion levels similar to those of the wild type strain.

Since one of *Brucella’s* main routes of infection is oral (mainly due to the intake of unpasteurized dairy products), the role of Bma proteins in the adhesion to cell types linked to the digestive tract was studied. The *bmaA* mutant showed a significant decrease in the adhesion to colon epithelial cells (HT-29 and Caco-2 cell lines), whereas the complementing plasmid restored the adhesive phenotype (**Figure 2C**). Absence of BmaB or BmaC did not affect the adhesion of *B. suis* to these cells.

*Brucella* spp. has been linked to abortion in animals and humans, and trophoblasts have been shown to be a preferred niche for bacterial survival and replication; thus, adhesion studies were also performed with a model trophoblastic cell line (Swan 71). In this case, the mutants did not show differences in comparison with the control strain. However, the complemented strains of *bmaA* and *bmaB* mutants showed a significant increase in adhesion (**Figure 2D**). This is probably due to a multicopy effect of the pBBR1-MCS1 plasmid expressing the *bmaA* or *bmaB* genes from their own promoters.

Our results showed that the *bma* mutants do not display the same adhesive phenotypes in all the cell types analyzed. These phenotypes could be due to compensatory effects on the expression of another *bma* gene. However, analysis of the expression of *bma* genes in the wild type and the *bma* mutants by RT-qPCR did not show differences in the mRNA levels (**Figure S2**).

Taken together, these results suggest that the classical autotransporters contribute to a greater or lesser extent to the initial adhesion of *B. suis* 1330 to different cell types involved in either the infection route or the pathological manifestations of brucellosis.

**Figure 3:**
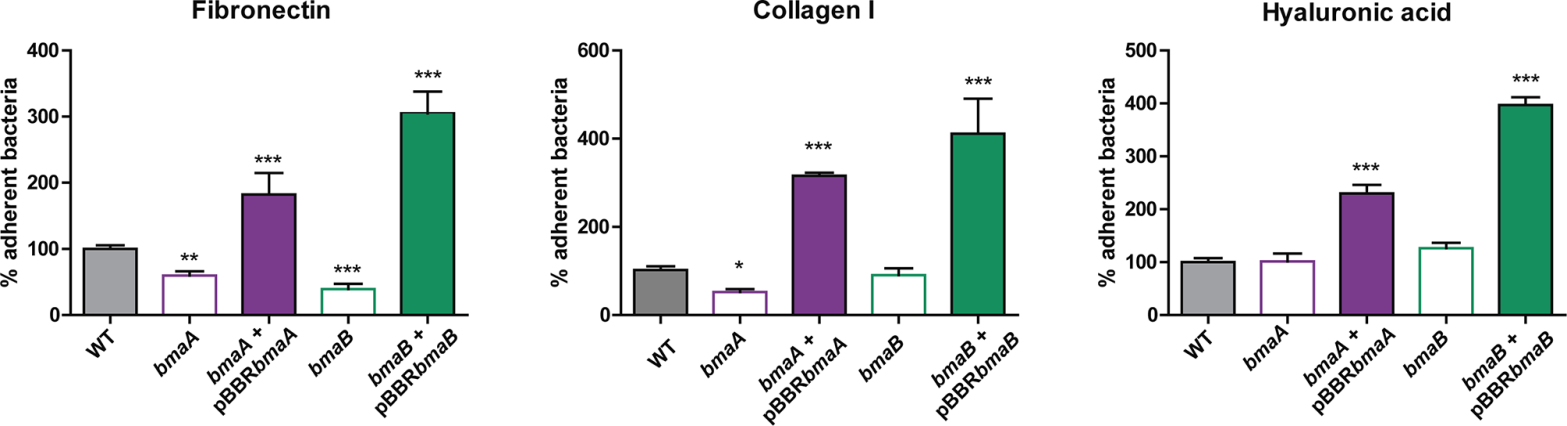
Adherence of *B. suis* strains to ECM components. Binding to immobilized ligands of *B. suis* wild type, *B. suis bmaA*::stops (*bmaA*), *B. suis bmaB*::Km (*bmaB*), and the respective complemented strains (*bmaA::*st + pBBR*bmaA, bmaB*::Km + pBBR*bmaB* and *bmaC* KI) was assayed. Values correspond to the percentage of binding to ECM components. A value of 100% was assigned to control strains. Data represent the means and standard deviations (SD) of the results of a representative experiment done in triplicate. Three independent experiments were performed with similar results. Data were analyzed by one-way ANOVA followed by a Tukey´s *post hoc* test. *, significantly different from control p < 0.05, **p < 0.005, ***p < 0.0001.

Many evidences suggest that *Brucella’*s adherence to ECM would be important for the infectious process (Castaneda-Roldan et al., 2004; Hernandez-Castro et al., 2008; Czibener and Ugalde, 2012; Posadas et al., 2012; Ruiz-Ranwez et al., 2013a; Ruiz-Ranwez et al., 2013b; De Figueiredo et al., 2015). Furthermore, it was previously shown that BmaC exhibits affinity for fibronectin (Posadas et al., 2012). To explore whether BmaA and BmaB are also involved in the adhesion of *B. suis* to ECM components, the parental and mutant strains were exposed to immobilized fibronectin, type I collagen, and hyaluronic acid (**Figure 3**). The *bmaA* and the *bmaB* mutants showed a 40 and 60% reduction, respectively, in the adhesion to fibronectin, in comparison with the wild type strain. On the other hand, the *bmaA* mutant showed a 48% decrease in the adhesion to type I collagen compared to the control strain, while the *bmaB* mutant did not vary significantly. None of the mutants presented a significant difference in the adhesion to hyaluronic acid compared to the control strain. Interestingly, the complemented strains showed higher levels (between 180 and 400%) of bacterial binding to fibronectin, type I collagen, and hyaluronic acid, compared with the wild type strain, indicating that there was a “gain of function” in the strains carrying *bmaA* and *bmaB* genes in the multicopy pBBR-MCS1 plasmid (**Figure 3**).

**Figure 4:**
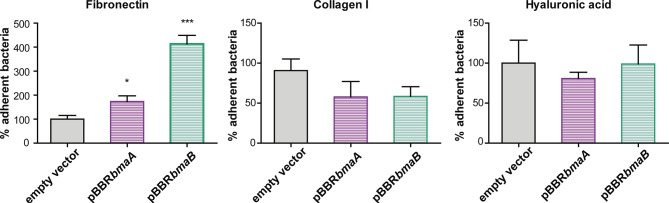
Evaluation of the role of BmaA and BmaB in the adherence to ECM components by heterologous expression. Binding to immobilized ligands of *E. coli* CC118 with an empty pBBR1-MCS1 (EV), *E. coli* CC118 pBBR*bmaA*, and *E. coli* CC118 pBBR*bmaB* was assayed. Values correspond to the percentage of binding to ECM components. A value of 100% was assigned to control strain. Data represent the means and standard deviations (SD) of the results of a representative experiment done in triplicate. Three independent experiments were performed with similar results. Data were analyzed by one-way ANOVA followed by a Tukey´s *post hoc* test. *, significantly different from control p < 0.05, ***p < 0.0001.

To further evaluate the ability of BmaA and BmaB to bind ECM components, a heterologous approach was carried out. The non-adherent *E. coli* CC118 strain was transformed with cloned *bmaA* or *bmaB* in pBBR-MCS1. This strategy has the advantage of providing evidence of adhesin activity that would otherwise be masked by redundant functions with other adhesins in the homologous bacteria. The pBBR-MCS1 plasmid carrying the *bmaA* or *bmaB* genes increased almost 1- and 4-fold, respectively, the adhesion of non-adherent *E. coli* cells to immobilized fibronectin compared with bacteria containing the empty vector (**Figure 4**). These results further support the role of Bma proteins in the binding to fibronectin.

The plasmids containing the *bmaA* or *bmaB* genes did not confer to *E. coli* the ability to adhere to immobilized type I collagen even though a slight decrease in the adhesion of the *B. suis bmaB* mutant was observed. In line with the phenotypes of the mutants, *E. coli* containing the pBBR*bmaA* or pBBR*bmaB* plasmids showed similar levels of adhesion to hyaluronic acid than the *E. coli* pBBR-MCS1 control strain (**Figure 4**).

Adhesins often contribute to bacterial adhesion to abiotic surfaces. By performing the classic biofilm assay on polystyrene plates and staining with crystal violet, we observed no differences in the attachment to polystyrene between the wild type and *bma* mutant strains of *B. suis* (**Figure S3**). Similarly, no differences in autoagglutination were observed between the mutants and the wild type strains at any time point during the whole follow-up period of 96 h (**Figures S4A, B**). To avoid interference between different adhesins we also evaluated autoagglutination in the non-adhesive *E. coli* CC118 strain carrying the pBBR*bmaA* or pBBR*bmaB* plasmids. However, again, no differences in the kinetics of autoaggregation were observed between the different strains during the whole follow-up period (**Figure S4C**). These observations suggest that, under the conditions tested, BmaA and BmaB appear not to be involved in adhesion to abiotic surfaces or in autoagglutination.

### Subcellular Localization

**Figure 5:**
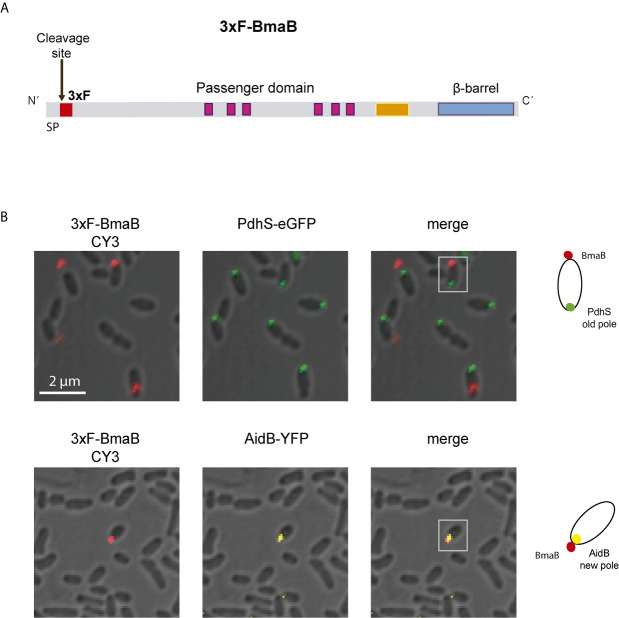
Polar localization of BmaB. **(A)** Schematic representation of the 3xFLAG tag in the BmaB protein. The FLAG (in red) was cloned immediately after the predicted cleavage site of the signal peptide. **(B)** Immunofluorescence assays with anti-FLAG antibodies were performed in *B. suis*-3xFLAG-BmaB expressing the pole markers PdhS-eGFP (upper panel) and AidB-YFP (lower panel). Bacterial shapes were observed by Differential Interference Contrast (DIC) images and cells were observed by confocal microscopy using a ZEISS LSM 880 Confocal Laser Scanning Microscope. In the first panel (from left to right) the label corresponding to BmaB is observed, in the second panel the label corresponding to the pole marker (PdhS or AidB) and in the third panel the merge of the previous ones. Gray squares indicate which bacteria are represented in the cell cartoons on the right that indicate the localization of BmaB and pole markers; red dots represent 3xFLAG-BmaB green and yellow intracellular dots represent PdhS and AidB localization (old and new pole respectively). The cartoons are based on the observations of this experiment and previous data on the localization of the pole markers AidB-YFP and PdhS-eGFP (Dotreppe et al., 2011; Van der Henst et al., 2012) and BmaB subcellular fractioning analysis (Bialer et al., 2019).

In order to interact with the host cell, either directly or through the ECM, autotransporter adhesins must be translocated and exposed on the cell surface. As mentioned earlier, it has been described that in *B. suis* the BmaC autotransporter has unipolar localization on the bacterial surface (Posadas et al., 2012). Moreover, it was proposed that this pole corresponds to the new pole generated after asymmetrical cell division (Ruiz-Ranwez et al., 2013a; Ruiz-Ranwez et al., 2013b). To evaluate whether other Bma adhesins also exhibit unipolar localization on the cell surface, immunofluorescence analysis using 3xFLAG-BmaB labeled strain of *B. suis* (Bialer et al., 2019) was performed (**Figure 5A**). Bacteria were fixed with 4% paraformaldehyde in PBS and analyzed by immunofluorescence using anti-FLAG antibodies and confocal microscopy. Under our assay conditions, BmaB was detected in 8% of bacteria examined. In all cases the red signal corresponding to BmaB showed unipolar location (**Figure 5B**).

In order to determine the cellular pole on which the adhesin is exposed, *B. suis* pBBR3xFLAG*bmaB* strain was conjugated with the pMR10cat replicative plasmid expressing AidB-YFP fusion protein as new pole marker (Dotreppe et al., 2011) or the PdhS-eGFP tagged protein, as old pole marker (Hallez et al., 2007). PdhS-eGFP was detected in 57% of bacteria (n = 616). In the cases where the red (3xFLAG-BmaB) and green (PdhS-GFP) foci were detected simultaneously (7%), they were found at opposite poles in 68% of the cases (**Figure 5B**). In the rest of cells in which both signals were detected, the red fluorescence corresponding to BmaB was observed either in the bacterial septum or in the same pole as PdhS. AidB-YFP was detected in 31% of the bacteria and only 2% of them showed both red (3xFLAG-BmaB) and yellow (AidB-YFP) fluorescence (n = 314). In all cases in which both signals were simultaneously detected, they were localized in the same pole. Thus, similar to the other autotransporter adhesins, BmaB appears to be expressed mostly at one particular bacterial pole and that pole corresponds to the new pole generated after cell division.

To test whether BmaB maintains its localization and function in the *B. suis* strain expressing the 3xFLAG tagged protein we performed the fibronectin binding assay. We observed that the wild type strain harboring the pBBR3xFLAG*bmaB* plasmid increases the levels of binding to fibronectin to similar levels to those of the complemented strain expressing *bmaB* (without the tag) from the multicopy plasmid (**Figure 3**), indicating that the tagged protein is correctly localized and is functional (**Figure S5**).

BmaB (and also BmaA but to a lesser extent) was able to confer to *E. coli* CC118 the ability to bind fibronectin (**Figure 4**). In order to evaluate the localization of BmaB in *E. coli*, we performed immunofluorescence assays with and without fixation using anti-FLAG primary antibody and Cy3 as secondary antibody. As expected, the 3xFLAG-BmaB signal was associated with the *E. coli* cell envelope. Interestingly, similar to that observed in *B. suis*, the protein localized mostly at one bacterial pole (**Figure S6**).

### *In Silico* Analysis of Bma Orthologues

**Figure 6:**
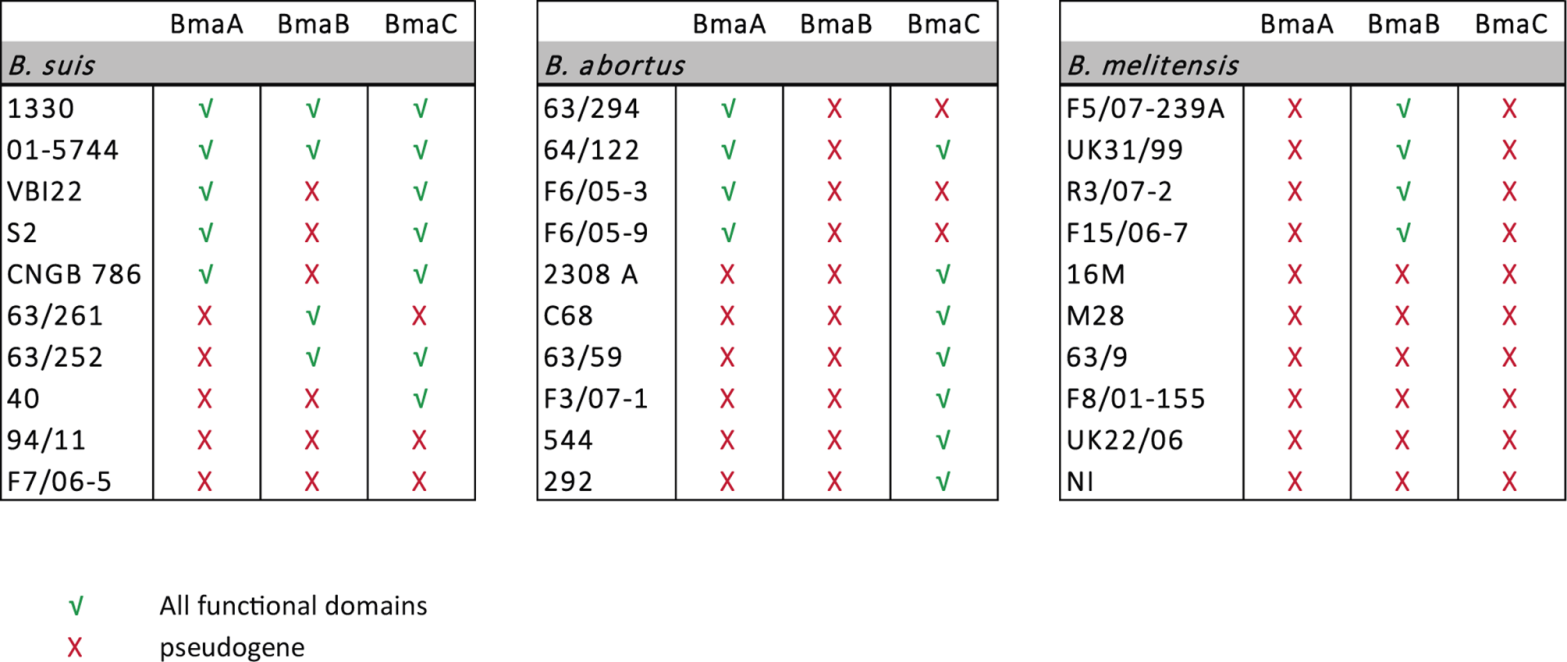
Protein analysis of type Va autotransporters in *Brucella* spp. Sequence analysis of BmaA, BmaB, and BmaC orthologues in *B. suis*, *B. abortus*, and *B. melitensis* strains. Red cross indicates pseudogene and green tick indicate that both the signal peptide and the β-barrel domain, which are required for proper translocation of the autotransporter through the inner and outer membranes are present.

Genomes of different *Brucella* species are strikingly similar to each other, displaying only a limited diversity that is likely responsible for differences in host preference, antigenic characteristics, and virulence. Interestingly, several of the few differences were found in genes encoding putative surface proteins such as outer membrane proteins (OMP) (Paulsen et al., 2002; Halling et al., 2005). Since these proteins may be involved in the initial interaction with the host, they are expected to be under high selective pressure. It could be proposed that genes encoding surface proteins undergo mutations throughout speciation events. Some of these mutations could alter domains/motifs or induce local structural changes; accumulation of mutations could even inactivate genes resulting in pseudogenization. As described above, the predicted BmaA and BmaB proteins from *B. suis* strain 1330 contain all the information needed to be functional, and *in vitro* studies confirmed that these proteins are involved in the adhesion of this strain to several human cell types. In contrast, it seems that the orthologous proteins from *B. abortus* strain 2308 and *B. melitensis* strain 16M would not be functional since they do not harbor all the essential domains for proper translocation to the cell surface. To assess whether these differences in Bma properties between *Brucella* species are restricted to reference or domesticated strains, the predicted Bma proteins from 30 strains of *B. suis*, *B. abortus*, and *B. melitensis* (10 each), whose genome sequences are deposited in the NCBI database, were analyzed looking for the presence of intact essential domains. The complete results are shown in **Table S2** and are summarized in **Figure 6**. All the genomes of the analyzed strains harbor the loci of the BmaA, BmaB, and BmaC orthologues. Furthermore, in all cases, the *bma* genes show a conserved synteny between species and strains. **Figure S7** shows the conserved synteny of the regions adjacent to *bmaA*, *bmaB*, and *bmaC* genes among the genomes of the *B. sui*s 1330, *B. abortus* 2308 and *B. melitensis* 16M reference strains. However, several genes show nonsense or frameshift mutations in their sequences, causing the interruption in translation and the consequent absence of one or both of the essential domains needed for protein secretion. In addition, the Bma autotransporters that were potentially functional show, in some cases, both inter- and intra-species differences in relation to their size, number of PATR repetitions and the presence or absence of adhesion-related domains (**Table S2**).

In half of the strains analyzed of *B. suis*, including strain 1330, BmaA seems to contain both the signal peptide and the C-terminal translocation domain. In the other orthologues, the locus splits into three ORFs due to the presence of premature stop codons, suggesting that they correspond to pseudogenes.

BmaA orthologues of *B. abortus* tend to be larger than those of *B. suis* strains (around twice the size) and with a higher number of PATR domains. The BmaA proteins of F6/05-3, F6/05-9, 63/294, and 64/122 strains exhibit all the essential domains to be correctly translocated to the cell surface, although the ORF would start 64 amino acids earlier than what is proposed in the annotated version. However, in the rest of the orthologues analyzed, the BmaA protein appears to be non-functional. BmaA from strains 2308 and F3/07-1 have a premature stop codon within the passenger domain and therefore do not harbor the C-terminal β-barrel, indicating than they probably correspond to pseudogenes. The *bmaA* gene of strain C68 has a premature stop codon at the N-terminal end of the protein causing the ORF to be divided into two; the larger ORF (that includes the C-terminal translocation domain) lacks the signal peptide, again suggesting that it would be a pseudogene. In the case of strains 63/59 and 544, the locus splits into three ORFs, suggesting they are all pseudogenes.

The *bmaA* loci of all strains analyzed of *B. melitensis* are likely to be pseudogenes. The locus splits into three or four predicted ORFs by different mutations that ultimately generate stop codons. One of these mutations is a conserved deletion of two bases (**Figure S8**), which generates a reading frame shift and a TAA stop codon.

In the case of BmaB orthologues of *B. suis*, in addition to the strain 1330, complete proteins were found in 01-5744, 63/252, and 63/261 strains. The predicted BmaB protein from the other strains, including those of biovar 2, may not be functional since they all have a mutation that causes them to lose the translocator C-terminal domain.

BmaB orthologues of *B. abortus* would be pseudogenes in all strains analyzed. In all cases the presence of a premature stop codon generates two truncated peptides. Another mutation generated a truncated ORF at the 5’ end of the gene that would start upstream of the annotated gene. These mutations have probably occurred in a common ancestor since they were conserved in all the *B. abortus* strains analyzed (**Figure S8**).

In four out of 10 analyzed strains of *B. melitensis* (F5/07-239A, UK31/99, R3/07-2, and F15/06-7) the *bmaB* gene encodes both a signal peptide and the translocator domain, suggesting that the autotransporters might be functional. In these cases, BmaB seems to be similar to its orthologues of *B. suis* regarding number of PATR repetitions and length. In the rest of the *B. melitensis* strains, the *bmaB* loci would be pseudogenes since they show a conserved mutation within the passenger domain. However, this mutation is not the same as the one detected in *bmaB* of *B. abortus*. The case of the 16M reference strain is particular since *bmaB* would be also a pseudogene but for a different reason; in this case the locus has a non-sense mutation very close to the N-terminal end, eliminating the signal peptide.

BmaC orthologues of *B. suis* and *B. abortus* are complete in several strains; in these cases, the proteins exhibit the same length and number of PATRs showing less variation amongst them. Mutations present in the *bmaC* locus of some *B. suis* strains (63/264, 94/11, and F7/06-5) are conserved giving rise to three truncated ORFs, while in *B. abortus* the mutations are randomly distributed. The *bmaC* orthologues of *B. melitensis* would be pseudogenes in all strains analyzed showing a conserved mutation in all cases.

According to the phylogeny of *Brucella* spp., *B. abortus* and *B. melitensis* are closer relatives between them than with *B. suis* (Foster et al., 2009). Since the mutations detected in some of the autotransporter loci of *B. abortus* and *B. melitensis* were conserved within each lineage but were different from each other, it can be proposed that they did not occur in the common ancestor that gave rise to speciation but occurred in parallel, suggesting convergent evolution (Merhej et al., 2009).

In conclusion, it seems that in *B. abortus* while BmaA and the BmaC proteins could play a role in several of the strains analyzed, the *bmaB* locus would not encode a functional protein in any case. Conversely, in *B. melitensis*, while the *bmaA and bmaC* loci are not functional, the BmaB autotransporter could potentially contribute to adherence to host components in several of the strains analyzed. *B. suis* would be an intermediate case since BmaA and/or BmaB and/or BmaC could be functional depending on the strain/biovar.

Taking together all these observations, it can be proposed that variations in the presence of functional type Va autotransporters amongst *Brucella* species/biovars are some of the strategies adopted by the pathogen to modulate its ability to interact with their species-specific or preferred host.

## Discussion

Bacteria of *Brucella* genus are facultative intracellular pathogens capable to infect a wide variety of cell types (Liautard et al., 1996; Billard et al., 2005; Delpino et al., 2009; Ferrero et al., 2009; Delpino et al., 2010; Scian et al., 2011; von Bargen et al., 2012; Fernandez et al., 2016). To invade cells, bacteria must be able to adhere to the cell surface. In fact, several adhesins have been shown to participate in adhesion and/or invasion to host cells, including the BmaC type Va autotransporter and the BtaE and BtaF trimeric autotransporters (Castaneda-Roldan et al., 2006; Czibener and Ugalde, 2012; Posadas et al., 2012; Ruiz-Ranwez et al., 2013a; Ruiz-Ranwez et al., 2013b; Czibener et al., 2016; Bialer et al., 2020; Lopez et al., 2020).

The genome of *B. suis* strain 1330 harbors the genes of two additional type Va autotransporters, BmaA and BmaB, that (although much smaller) share relatively high similarity with BmaC. The observations described in this work indicate that in *B. suis* 1330, BmaA and BmaB contribute to bacterial adhesion to different cell types and ECM components.

It was shown that smooth strains of *B. abortus*, *B. melitensis*, and *B. suis* are capable of invading colon epithelial cells such as HT-29 and Caco-2, and replicate there efficiently (Delpino et al., 2009; Ferrero et al., 2012). Our results indicate that BmaA could be involved in the adhesion of *B. suis* 1330 to these cell types. The digestive tract is considered to play a fundamental role in the oral route of infection (Tsolis et al., 2008; Ferrero et al., 2012). The importance of the *virB* operon, urease enzyme, bile acid hydrolases, and the LPS O-antigen in the establishment of infection by the oral route has been proven (Delpino et al., 2007; Paixao et al., 2009). However, little is known about the mechanisms that mediate the initial contact of bacteria with cells of the digestive tract. In *B. abortus* two adhesins with immunoglobulin-like domains (BigA and BigB) were shown to contribute to bacterial adhesion to polarized intestinal epithelial cells such as Caco-2. Interestingly, addition of recombinant BigA or BigB proteins to cells induces cytoskeleton rearrangements and affects focal adhesion sites. The authors proposed that the Big adhesins target cell-cell junctions or ECM proteins (Czibener et al., 2016; Lopez et al., 2020). One possibility is that both Big and Bma adhesins could play complementary roles in adhesion to epithelial cells either directly or through ECM components.

A prevalent manifestation of human brucellosis is osteoarticular involvement, which is present in 27 to 36% of brucellosis patients (Adetunji et al., 2019). Naturally smooth *Brucella* species are capable of invading and replicating in human osteoblasts (Delpino et al., 2009). In addition, it was shown that *Brucella* spp. can infect human synoviocytes and induce the secretion of matrix metalloproteases that might be involved in the osteoarticular manifestations of brucellosis (Scian et al., 2011; Scian et al., 2013). Here we showed that the mutants that lack BmaA, BmaB, or BmaC adhesins are impaired in the adhesion of *B. suis* 1330 to both an osteoblastic cell line and primary osteoblast cultures as well as to a synoviocyte cell line and synoviocyte primary cultures. These observations suggest that the type Va autotransporters might play a role in the tropism of *B. suis* to the osteoarticular tissue.

*Brucella* has a pronounced tropism towards animal reproductive organs generating orchitis, epididymitis, and infertility in males, and abortions and sterility in females (Godfroid et al., 2005; Pappas et al., 2006). In humans, obstetric complications such as abortions and preterm delivery have been also observed (Sarram et al., 1974; Lulu et al., 1988; Bosilkovski et al., 2019; Liu et al., 2020). Genital secretions and abortion products contain a large bacterial load and constitute an important source of infection among animals. Therefore, it is clear that replication in the reproductive tract of these animals is crucial for *Brucella*’s biology. In fact, it has been shown that *Brucella* can invade and replicate in trophoblasts (Sarram et al., 1974; Lulu et al., 1988; Salcedo et al., 2013; Fernandez et al., 2016). Our results indicate that, although the *bma* mutants were not impaired in adhesion to trophoblasts, expression of the *bmaA* and *bmaB* genes from a multi-copy plasmid conferred a significant increase in adhesion to these cells, suggesting that BmaA and BmaB might play a role in the adhesion of *B. suis* to trophoblasts. As expected, a similar effect was not observed with the *bmaC* complemented strain since this strain was generated by *knock-in* (the mutated gene was replaced in the genome by the wild type sequence) and not by *bmaC* expression from a multi-copy plasmid. Possible explanations for the absence of mutant phenotypes in the adhesion to trophoblasts might be that 1) Bma proteins have redundant adhesive functions, so that knocking out just one does not impair binding to these cells, 2) other surface factors provide a redundant role able to overcome the lack of Bma proteins, and 3) *in vitro* and in these model cells, the Bma adhesins are not fulfilling an active role. In the latter case, however, Bma functions become evident in a “gain of function” genetic context; i.e., under our assay conditions the endogenous expression of the Bma adhesins is not sufficient to observe an adhesion phenotype to trophoblasts, and other host environmental stimuli are necessary to mimic physiological conditions.

The results of cell adhesion studies suggest that the relevance of a particular Bma adhesin for *Brucella* binding varies according to the cell type. This is probably related to the repertoire and/or abundance of adhesin ligands on the surface of the different cell types. Besides, it seems that the Bma proteins have overlapping functions in terms of ligands to which they bind. This redundancy may contribute to explain why the deletion or inactivation of a particular *bma* gene usually results in only a partial reduction of bacterial adhesion. There is evidence suggesting that *Brucella* spp. binds to components of the ECM, in particular fibronectin, hyaluronic acid, collagen, and molecules rich in sialic acid (Campbell et al., 1994; Castaneda-Roldan et al., 2004; Posadas et al., 2012; Ruiz-Ranwez et al., 2013a; Ruiz-Ranwez et al., 2013b; ElTahir et al., 2019). The mutant phenotypes suggest that both BmaA and BmaB contribute to the adhesion of *B. suis* to fibronectin, while only BmaA may contribute to the binding of *B. suis* to type I collagen. However, *bma* expression from a multi-copy plasmid produced a significant increase in the adhesion to all the ECM components analyzed, suggesting that BmaA and BmaB fulfill an active role in binding to ECM components.

Experiments of heterologous expression in *E. coli* support a role of BmaA and BmaB in the adhesion to fibronectin while no differences compared with the control were observed in the binding of *E. coli* that harbors the pBBR*bmaA* plasmid to type I collagen. Since both the *bmaA* and *bmaB* transcripts were equally detected by RT-PCR in the heterologous context of *E. coli* (**Figure S6B**), the possibility exists that BmaA is not capable of fulfilling the same roles in a surrogate bacterium.

The role of BmaA and BmaB in adhesion to fibronectin is not surprising since we have previously demonstrated that BmaC, which shares significant similarities with BmaA and BmaB, is involved in adhesion of *B. suis* 1330 to HeLa and A549 epithelial cells through the binding to cellular fibronectin (Posadas et al., 2012). It seems that *Brucella* type Va autotransporters fulfill redundant functions or, alternatively, they are differentially expressed in different host tissues/niches. Besides, the possibility exists that Bma proteins bind to other molecules (not evaluated in this work) on the surface of target cells, in addition to the ECM. It is worth mentioning that although binding assays using cultured cells provide valuable information to understand the role of bacterial adhesion factors, complete expression of the adhesins might be dependent on additional environmental stimuli. For instance, the expression of adhesin genes could be induced in contact with cells but in the context of certain host tissues. Future studies will be necessary to elucidate if the type Va (monomeric) and type Vc (trimeric) autotransporter adhesins work together under the same environmental conditions or, instead, they are expressed under different stimuli, fulfilling complementary roles. Furthermore, double or triple mutants could be generated to evaluate whether two or more adhesins contribute jointly to the binding to a specific ECM ligand or cell type.

We found that BmaB is detected at one particular bacterial pole. Using fluorescent markers, we could observe that this pole corresponds in most cases to the new pole generated after asymmetric cell division. Similar observations were described for BmaC, BtaE, and BtaF (Posadas et al., 2012; Ruiz-Ranwez et al., 2013a; Ruiz-Ranwez et al., 2013b), supporting the hypothesis that the new pole generated after cell division would be functionally differentiated for adhesion (Van der Henst et al., 2013). Other proteins from the monomeric type Va autotransporter family, such as IcsA of *Shigella flexneri* (Goldberg et al., 1993) and the AIDA-I adhesin from *E. coli* (Jain et al., 2006) also show unipolar localization. It is interesting that these surface proteins showed polar localization even in the cytoplasm, prior to its secretion, suggesting not only that protein secretion would be polarly localized but also that the mechanism that defines the polarity is cytoplasmic (Steinhauer et al., 1999; Jain et al., 2006). Interestingly, BmaB showed unipolar localization not only in *B. suis* but also when it was expressed in *E. coli*, as observed previously for BtaE and BtaF (Ruiz-Ranwez et al., 2013a; Ruiz-Ranwez et al., 2013b), suggesting that the mechanisms that direct the translocation and polar localization of autotransporters are conserved in different bacteria. It would be interesting to perform further studies to elucidate if there is a level of substrate specificity of TAM and/or BAM translocation systems in *E. coli* and *Brucella*.

Although the construction expressing 3xFLAG-BmaB was designed in a way that the *bmaB* gene is expressed from the *lac* promoter, the fluorescence foci corresponding to BmaB were detected in only 8% of cells. We have previously shown that BmaC, BtaE and BtaF are also expressed (from their own promoters) in a low number of bacteria. Therefore, an adhesive pole that is expressed in a small but infective cell subpopulation was previously proposed (Ruiz-Ranwez et al., 2013a; Ruiz-Ranwez et al., 2013b). In fact, a particular cell type (consisting of infectious “newborn” cells) is the predominant cell type of the *B. abortus* cell cycle found just after internalization into the host cell (Deghelt et al., 2014). Polar adhesin expression in a small bacterial subpopulation could be sufficient to achieve *Brucella* internalization and thus establish a limited but successful infection. Hence, the question is how an adhesive pole is regulated in a particular and infective bacterial cell type. This could be achieved not only by gene expression regulation, but also by a fine tuning of adhesin expression including post-transcriptional or post-translational mechanisms as in the case of *E. coli* AIDA-I autotransporter (Charbonneau et al., 2007). Furthermore, the regulation of the repertoire of adhesins could also come from the interplay between these OMPs and the BAM and TAM translocation machineries.

Several surface factors, including adhesive structures of other *Alphaproteobacteria* phylogenetically related to *Brucella*, exhibit polar localization. It was described that proteins of the VirB secretion system from *Agrobacterium tumefaciens* have polar localization (Judd et al., 2005). Both, *Rhodopseudomonas palustris* and *A. tumefaciens* are capable of adhering to plants and abiotic surfaces through one of their poles, in a process mediated by different structures, such as the unipolar polysaccharide adhesin (UPP) (Tomlinson and Fuqua, 2009). Another example of an *Alphaproteobacteria* that has a polar architecture is *Caulobacter crescentus*; in this case the old pole generated after cell division is the one differentiated for adhesion (Skerker and Laub, 2004; Tomlinson and Fuqua, 2009; Brown et al., 2012; Kirkpatrick and Viollier, 2012). In summary, in *Brucella*, an attractive hypothesis is that the initial adhesion to the host cell would be mediated by adhesins localized to the new (adhesive) pole and that their expression occurs only in an infectious bacterial subpopulation.

Besides *B. suis* 1330, also the genomes of the *B. abortus* 2308 and *B. melitensis* 16M reference strains show the presence of both the *bmaA* and *bmaB* loci, although in these cases they seem to correspond to pseudogenes. In addition, the predicted large BmaC protein is complete in *B. abortus* 2308 (and *B. suis* 1330) but the corresponding locus would be formed by pseudogenes in *B. melitensis* strain 16M. To investigate whether these observations are general features of the *bma* genes from the different species or biovars, we carefully analyzed the orthologous loci of numerous strains of *B. suis*, *B. abortus*, and *B. melitensis*. All the strains analyzed not only harbor all three *bma* loci, but also show a conserved synteny (**Figure S7**). However, we observed that for all the *B. abortus* strains analyzed, the *bmaB* locus is consistently made up of pseudogenes, while the BmaA and BmaC adhesins could still be functional in several strains/biovars. Conversely, in *B. melitensis*, while both the *bmaA* and *bmaC* loci are made up of pseudogenes in all strains analyzed, the BmaB autotransporter could still play a role in adhesion in several strains. Surprisingly, in contrast with BmaA, BmaB, and BmaC adhesins from *B. suis* 1330 strain, the predicted *bma* orthologues from some *B. suis* strains seem to encode truncated proteins. Nevertheless, in most cases the *B. suis* genomes harbor at least two Bma autotransporters that could be functional (**Table S2**). The predicted BmaA proteins of *B. abortus* are larger and with higher number of PATR motifs than those of *B. suis*. In contrast, the predicted (and potentially functional) BmaB proteins of *B. melitensis* were similar in size and repetition numbers compared with those of *B. suis*.

*In silico* analysis clearly showed that within the *bmaB* locus of most strains analyzed of *B. abortus* and the *bmaA* and *bmaC* loci of *B. melitensis*, two or three non-sense mutations occurred, giving rise to pseudogenes. These mutations were highly conserved intra-species, suggesting that these loci were no longer functional during early stages of speciation. Since mutations responsible for pseudogenization of the classical autotransporter loci are not conserved between species, it can be proposed that they did not occur in a common ancestor. It seems that the mutational events developed in parallel in the three lineages, resulting in some cases a clear example of convergent evolution (Merhej et al., 2009). It is possible that the expression of either functional adhesins or the generation of pseudogenes is due to the selection pressure on bacterial surface proteins, which are in permanent contact with the host’s changing environment (Paulsen et al., 2002; Ferreira et al., 2017).

Within the *Brucella* genus the concept of species is particularly controversial (Moreno et al., 2002), mainly due to the high genome conservation (Rajashekara et al., 2004; O’Callaghan and Whatmore, 2011). This has led some authors to propose that *Brucella* species correspond in fact to biovars or strains of a single species (Corbel, 1983; Verger, 1985; Gandara et al., 2001). This theory has been dismissed mainly because it underestimates the importance of genetic divergence in explaining the differences between organisms in relation to host preferences (Moreno, 2001; Ficht, 2010). Interestingly, comparison of the genomes of *B. abortus* 2308, *B. suis* 1330 and different strains of *B. suis* bv. 2 indicated that the most variable genes largely encode hypothetical proteins, many of which would correspond to surface proteins (Paulsen et al., 2002; Chain et al., 2005; Ferreira et al., 2017). Particularly, proteins from the autotransporter families were associated with speciation events. Besides, genes that encode these proteins in *Brucella* exhibit an atypical nucleotide composition, suggesting that they were acquired by lateral gene transfer (Paulsen et al., 2002; Rajashekara et al., 2004; Chain et al., 2005; Halling et al., 2005). These events, followed by selection pressure depending on the host environment, can elicit differential virulence factors that contribute to generate new species/biovars. Our *in silico* observations reinforce the hypothesis that type Va autotransporters could constitute a group of adhesins that are differentially expressed in different *Brucella* species or biovars, and therefore could contribute to host preferences. However, this possibility needs to be further explored.

The significant sequence similarities between *bmaA*, *bmaB*, and *bmaC* (approximately 40% in *B. suis* 1330) suggest that they are paralogs. Besides, comparison between the *bma* full orthologues from the different species revealed a high degree of sequence conservation (greater than 98% of identity). Therefore, the duplication events of these genes must have occurred prior to speciation and the generation of pseudogenes would be an evolutionarily closer event.

It is worth mentioning that the lack of prediction of an N-terminal signal peptide in the autotransporter proteins annotated in the genomes was not considered as a criterion to define the presence of a pseudogene. This is because, in several cases, alternative start codons upstream of the annotated ORF were identified and the translation product of the new ORF contained an N-terminal signal peptide with a reliable score. The new proposed initiation codon was often different from the commonly used (ATG); in bacteria, GTG and TTG, and to a lesser extent CTG, are frequently used as alternative initiation codons (McCarthy and Brimacombe, 1994; Higgs and Ran, 2008). These observations indicate that protein annotations should be carefully analyzed when conducting both *in silico* and biochemical studies.

Although in several cases the *bma* loci would be pseudogenes, RT-PCR and RT-qPCR analyses detected *bmaA*, *bmaB*, and *bmaC* transcripts in *B. suis* 1330, *B. abortus* 2308, and *B. melitensis* 16M strains (data not shown), suggesting that there is not a negative regulation at promoter level that leads to the interruption of transcription of these loci. In fact, it has been described in *Mycobacterium leprae* that the majority of the pseudogenes are transcribed, but they seem to be regulated at a translational level by a variety of “silencing” mechanisms, reducing additional energy and resource expenditure required for protein production from the vast majority of these transcripts (Williams et al., 2009).

Taken together, our results show that BmaA, BmaB, and BmaC contribute, to a greater or lesser degree, to the adhesion of *B. suis* 1330 to different human cell types as well as to ECM components. The different species of *Brucella* display extensive variations in the repertoire of functional Bma autotransporters that can be exposed on the bacterial surface, supporting the hypothesis that diversity within this family of adhesins might be involved in tropism to certain tissues and in host preferences.

## Data Availability Statement

The raw data supporting the conclusions of this article will be made available by the authors, without undue reservation.

## Author Contributions

MGB, MCF, MVD, VR-R, DMP, PCB, and AZ have designed the experiments. MGB, MCF, MVD, DMP, and VR-R performed the experiments. MGB and AZ participated in draft preparation. MGB, MCF, MVD, DMP, VR-R, PCB, and AZ participated in the revision. All authors contributed to the article and approved the submitted version.

## Funding

PICT 2016-1199 and PICT 2016-2722 from Agencia Nacional de Promoción Científica (ANPCYT) and UBACYT 20020170100036BA from Universidad de Buenos Aires.

## Conflict of Interest

The authors declare that the research was conducted in the absence of any commercial or financial relationships that could be construed as a potential conflict of interest.

## References

[B1] Adetunji S. A. Ramirez G. Foster M. J. Arenas-Gamboa A. (2019). A Systematic Review and Meta-Analysis of the Prevalence of Osteoarticular Brucellosis. PloS Negl. Trop. Dis. 13, e0007112. 10.1371/journal.pntd.0007112 30657765 PMC6355028

[B2] Almagro Armenteros J. J. Tsirigos K. D. Sonderby C. K. Petersen T. N. Winther O. Brunak S.. (2019). SignalP 5.0 Improves Signal Peptide Predictions Using Deep Neural Networks. Nat. Biotechnol. 37, 420–423. 10.1038/s41587-019-0036-z 30778233

[B3] Artimo P. Jonnalagedda M. Arnold K. Baratin D. Csardi G. De Castro E.. (2012). ExPASy: SIB Bioinformatics Resource Portal. Nucleic Acids Res. 40, W597–W603. 10.1093/nar/gks400 22661580 PMC3394269

[B5] Bachvarov B. Kirilov K. Ivanov I. (2008). Codon Usage in Prokaryotes. Biotechnol. Biotechnol. Equip. 22, 669–682. 10.1080/13102818.2008.10817533

[B6] Bandara A. B. Sriranganathan N. Schurig G. G. Boyle S. M. (2005). Putative Outer Membrane Autotransporter Protein Influences Survival of Brucella Suis in BALB/c Mice. Vet. Microbiol. 109, 95–104. 10.1016/j.vetmic.2005.05.012 15970403

[B9] Benz I. Schmidt M. A. (1992). AIDA-I, the Adhesin Involved in Diffuse Adherence of the Diarrhoeagenic *Escherichia coli* Strain 2787 (O126:H27), is Synthesized Via a Precursor Molecule. Mol. Microbiol. 6, 1539–1546. 10.1111/j.1365-2958.1992.tb00875.x 1625582

[B10] Benz I. Schmidt M. A. (2011). Structures and Functions of Autotransporter Proteins in Microbial Pathogens. Int. J. Med. Microbiol. 301, 461–468. 10.1016/j.ijmm.2011.03.003 21616712

[B11] Bialer M. G. Ruiz-Ranwez V. Sycz G. Estein S. M. Russo D. M. Altabe S.. (2019). MapB, the Brucella Suis TamB Homologue, is Involved in Cell Envelope Biogenesis, Cell Division and Virulence. Sci. Rep. 9, 2158. 10.1038/s41598-018-37668-3 30770847 PMC6377625

[B12] Bialer M. G. Sycz G. Munoz Gonzalez F. Ferrero M. C. Baldi P. C. Zorreguieta A. (2020). Adhesins of *Brucella*: Their Roles in the Interaction With the Host. Pathogens 9 (11), 942. 10.3390/pathogens9110942 PMC7697752 33198223

[B14] Billard E. Cazevieille C. Dornand J. Gross A. (2005). High Susceptibility of Human Dendritic Cells to Invasion by the Intracellular Pathogens *Brucella suis*, *B. abortus*, and *B. melitensis* . Infect. Immun. 73, 8418–8424. 10.1128/IAI.73.12.8418-8424.2005 16299342 PMC1307067

[B15] Bosilkovski M. Arapovic J. Keramat F. (2020). Human brucellosis in pregnancy - an overview. Bosn. J. Basic Med. Sci. 20, 415–422. 10.17305/bjbms.2019.4499 31782698 PMC7664790

[B16] Brown P. J. De Pedro M. A. Kysela D. T. Van Der Henst C. Kim J. De Bolle X.. (2012). Polar Growth in the Alphaproteobacterial Order Rhizobiales. Proc. Natl. Acad. Sci. U. S. A. 109, 1697–1701. 10.1073/pnas.1114476109 22307633 PMC3277149

[B17] Buzgan T. Karahocagil M. K. Irmak H. Baran A. I. Karsen H. Evirgen O.. (2010). Clinical Manifestations and Complications in 1028 Cases of Brucellosis: A Retrospective Evaluation and Review of the Literature. Int. J. Infect. Dis. 14, e469–e478. 10.1016/j.ijid.2009.06.031 19910232

[B18] Campbell G. A. Adams L. G. Sowa B. A. (1994). Mechanisms of Binding of *Brucella abortus* to Mononuclear Phagocytes From Cows Naturally Resistant or Susceptible to Brucellosis. Vet. Immunol. Immunopathol. 41, 295–306. 10.1016/0165-2427(94)90103-1 7941309

[B19] Castaneda-Roldan E. I. Avelino-Flores F. Dall’agnol M. Freer E. Cedillo L. Dornand J.. (2004). Adherence of *Brucella* to Human Epithelial Cells and Macrophages is Mediated by Sialic Acid Residues. Cell Microbiol. 6, 435–445. 10.1111/j.1462-5822.2004.00372.x 15056214

[B20] Castaneda-Roldan E. I. Ouahrani-Bettache S. Saldana Z. Avelino F. Rendon M. A. Dornand J.. (2006). Characterization of SP41, a Surface Protein of *Brucella* Associated With Adherence and Invasion of Host Epithelial Cells. Cell Microbiol. 8, 1877–1887. 10.1111/j.1462-5822.2006.00754.x 16817909

[B21] Celik N. Webb C. T. Leyton D. L. Holt K. E. Heinz E. Gorrell R.. (2012). A Bioinformatic Strategy for the Detection, Classification and Analysis of Bacterial Autotransporters. PloS One 7, e43245–e43245. 10.1371/journal.pone.0043245 22905239 PMC3419190

[B22] Chain P. S. Comerci D. J. Tolmasky M. E. Larimer F. W. Malfatti S. A. Vergez L. M.. (2005). Whole-Genome Analyses of Speciation Events in Pathogenic *Brucellae* . Infect. Immun. 73, 8353–8361. 10.1128/IAI.73.12.8353-8361.2005 16299333 PMC1307078

[B23] Charbonneau M. E. Girard V. Nikolakakis A. Campos M. Berthiaume F. Dumas F.. (2007). O-Linked Glycosylation Ensures the Normal Conformation of the Autotransporter Adhesin Involved in Diffuse Adherence. J. Bacteriol. 189, 8880–8889. 10.1128/JB.00969-07 17951390 PMC2168594

[B25] Czibener C. Merwaiss F. Guaimas F. Del Giudice M. G. Serantes D. A. Spera J. M.. (2016). Biga is a Novel Adhesin of *Brucella* That Mediates Adhesion to Epithelial Cells. Cell Microbiol. 18, 500–513. 10.1111/cmi.12526 26400021

[B26] Czibener C. Ugalde J. E. (2012). Identification of a Unique Gene Cluster of *Brucella* Spp. That Mediates Adhesion to Host Cells. Microbes Infect. 14, 79–85. 10.1016/j.micinf.2011.08.012 21911075 PMC3247657

[B27] De Figueiredo P. Ficht T. A. Rice-Ficht A. Rossetti C. A. Adams L. G. (2015). Pathogenesis and Immunobiology of Brucellosis: Review of *Brucella*-host Interactions. Am. J. Pathol. 185, 1505–1517. 10.1016/j.ajpath.2015.03.003 25892682 PMC4450313

[B29] Deghelt M. Mullier C. Sternon J. F. Francis N. Laloux G. Dotreppe D.. (2014). G1-Arrested Newborn Cells are the Predominant Infectious Form of the Pathogen *Brucella abortus* . Nat. Commun. 5, 4366. 10.1038/ncomms5366 25006695 PMC4104442

[B30] Delpino M. V. Barrionuevo P. Scian R. Fossati C. A. Baldi P. C. (2010). *Brucella*-Infected Hepatocytes Mediate Potentially Tissue-Damaging Immune Responses. J. Hepatol. 53, 145–154. 10.1016/j.jhep.2010.02.028 20452697

[B31] Delpino M. V. Fossati C. A. Baldi P. C. (2009). Proinflammatory Response of Human Osteoblastic Cell Lines and Osteoblast-Monocyte Interaction Upon Infection With *Brucella* Spp. Infect. Immun. 77, 984–995. 10.1128/IAI.01259-08 19103778 PMC2643642

[B32] Delpino M. V. Marchesini M. I. Estein S. M. Comerci D. J. Cassataro J. Fossati C. A.. (2007). A Bile Salt Hydrolase of *Brucella abortus* Contributes to the Establishment of a Successful Infection Through the Oral Route in Mice. Infect. Immun. 75, 299–305. 10.1128/IAI.00952-06 17088355 PMC1828384

[B33] Dotreppe D. Mullier C. Letesson J. J. De Bolle X. (2011). The Alkylation Response Protein AidB is Localized At the New Poles and Constriction Sites in *Brucella abortus* . BMC Microbiol. 11, 257. 10.1186/1471-2180-11-257 22111948 PMC3236019

[B34] Doyle M. T. Tran E. N. Morona R. (2015). The Passenger-Associated Transport Repeat Promotes Virulence Factor Secretion Efficiency and Delineates a Distinct Autotransporter Subtype. Mol. Microbiol. 97, 315–329. 10.1111/mmi.13027 25869731

[B35] Drobnak I. Braselmann E. Chaney J. L. Leyton D. L. Bernstein H. D. Lithgow T.. (2015). Of Linkers and Autochaperones: An Unambiguous Nomenclature to Identify Common and Uncommon Themes for Autotransporter Secretion. Mol. Microbiol. 95, 1–16. 10.1111/mmi.12838 25345653 PMC4275399

[B36] Eltahir Y. Al-Araimi A. Nair R. R. Autio K. J. Tu H. Leo J. C.. (2019). Binding of *Brucella* Protein, Bp26, to Select Extracellular Matrix Molecules. BMC Mol. Cell Biol. 20, 55. 10.1186/s12860-019-0239-7 31783731 PMC6884894

[B37] Emsley P. Charles I. G. Fairweather N. F. Isaacs N. W. (1996). Structure of *Bordetella pertussis* Virulence Factor P.69 Pertactin. Nature 381, 90–92. 10.1038/381090a0 8609998

[B38] Eskra L. Canavessi A. Carey M. Splitter G. (2001). *Brucella abortus* Genes Identified Following Constitutive Growth and Macrophage Infection. Infect. Immun. 69, 7736–7742. 10.1128/IAI.69.12.7736-7742.2001 11705955 PMC98869

[B39] Fernandez A. G. Ferrero M. C. Hielpos M. S. Fossati C. A. Baldi P. C. (2016). Proinflammatory Response of Human Trophoblastic Cells to *Brucella abortus* Infection and Upon Interactions With Infected Phagocytes. Biol. Reprod. 94, 48. 10.1095/biolreprod.115.131706 26792938

[B41] Ferreira A. C. Tenreiro R. De Sa M. I. C. Dias R. (2017). Evolution and Genome Specialization of *Brucella suis* Biovar 2 Iberian Lineages. BMC Genomics 18, 726. 10.1186/s12864-017-4113-8 28899413 PMC5596481

[B42] Ferrero M. C. Fossati C. A. Baldi P. C. (2009). Smooth *Brucella* Strains Invade and Replicate in Human Lung Epithelial Cells Without Inducing Cell Death. Microbes Infect. 11, 476–483. 10.1016/j.micinf.2009.01.010 19397873

[B43] Ferrero M. C. Fossati C. A. Rumbo M. Baldi P. C. (2012). *Brucella* Invasion of Human Intestinal Epithelial Cells Elicits a Weak Proinflammatory Response But a Significant CCL20 Secretion. FEMS Immunol. Med. Microbiol. 66, 45–57. 10.1111/j.1574-695X.2012.00985.x 22553918

[B44] Ficht T. (2010). Brucella Taxonomy and Evolution. Future Microbiol. 5, 859–866. 10.2217/fmb.10.52 20521932 PMC2923638

[B46] Finn R. D. Bateman A. Clements J. Coggill P. Eberhardt R. Y. Eddy S. R.. (2014). Pfam: The Protein Families Database. Nucleic Acids Res. 42, D222–D230. 10.1093/nar/gkt1223 24288371 PMC3965110

[B47] Foster J. T. Beckstrom-Sternberg S. M. Pearson T. Beckstrom-Sternberg J. S. Chain P. S. Roberto F. F.. (2009). Whole-Genome-Based Phylogeny and Divergence of the Genus *Brucella* . J. Bacteriol. 191, 2864–2870. 10.1128/JB.01581-08 19201792 PMC2668414

[B49] Gandara B. Merino A. L. Rogel M. A. Martinez-Romero E. (2001). Limited Genetic Diversity of *Brucella* Spp. J. Clin. Microbiol. 39, 235–240. 10.1128/JCM.39.1.235-240.2001 11136777 PMC87708

[B50] Giambartolomei G. H. Delpino M. V. (2019). Immunopathogenesis of Hepatic *Brucellosis* . Front. Cell Infect. Microbiol. 9, 423. 10.3389/fcimb.2019.00423 31956605 PMC6951397

[B51] Corbel M. J. Gill K. P. W. Thomas E. L. Hendry D. Mc L. F. D. (1983). Methods for the identification of Brucella. (Alnwick, UK: MAFF Publications)

[B52] Godfroid J. Cloeckaert A. Liautard J. P. Kohler S. Fretin D. Walravens K.. (2005). From the Discovery of the Malta Fever’s Agent to the Discovery of a Marine Mammal Reservoir, *Brucellosis* has Continuously Been a Re-Emerging Zoonosis. Vet. Res. 36, 313–326. 10.1051/vetres:2005003 15845228

[B53] Goldberg M. B. Barzu O. Parsot C. Sansonetti P. J. (1993). Unipolar Localization and ATPase Activity of IcsA, a Shigella Flexneri Protein Involved in Intracellular Movement. J. Bacteriol. 175, 2189–2196. 10.1128/JB.175.8.2189-2196.1993 8468279 PMC204503

[B54] Hallez R. Mignolet J. Van Mullem V. Wery M. Vandenhaute J. Letesson J. J.. (2007). The Asymmetric Distribution of the Essential Histidine Kinase PdhS Indicates a Differentiation Event in *Brucella abortus* . EMBO J. 26, 1444–1455. 10.1038/sj.emboj.7601577 17304218 PMC1817626

[B55] Halling S. M. Peterson-Burch B. D. Bricker B. J. Zuerner R. L. Qing Z. Li L. L.. (2005). Completion of the Genome Sequence of *Brucella abortus* and Comparison to the Highly Similar Genomes of *Brucella melitensis* and *Brucella suis* . J. Bacteriol. 187, 2715–2726. 10.1128/JB.187.8.2715-2726.2005 15805518 PMC1070361

[B56] Hernandez-Castro R. Verdugo-Rodriguez A. Puente J. L. Suarez-Guemes F. (2008). The BMEI0216 Gene of *Brucella melitensis* is Required for Internalization in HeLa Cells. Microb. Pathog. 44, 28–33. 10.1016/j.micpath.2007.08.008 17881185

[B57] Higgs P. G. Ran W. (2008). Coevolution of Codon Usage and tRNA Genes Leads to Alternative Stable States of Biased Codon Usage. Mol. Biol. Evol. 25, 2279–2291. 10.1093/molbev/msn173 18687657

[B59] Jain S. Van Ulsen P. Benz I. Schmidt M. A. Fernandez R. Tommassen J.. (2006). Polar Localization of the Autotransporter Family of Large Bacterial Virulence Proteins. J. Bacteriol. 188, 4841–4850. 10.1128/JB.00326-06 16788193 PMC1483012

[B60] Judd P. K. Kumar R. B. Das A. (2005). The Type IV Secretion Apparatus Protein VirB6 of Agrobacterium Tumefaciens Localizes to a Cell Pole. Mol. Microbiol. 55, 115–124. 10.1111/j.1365-2958.2004.04378.x 15612921

[B61] Kirkpatrick C. L. Viollier P. H. (2012). Reflections on a Sticky Situation: How Surface Contact Pulls the Trigger for Bacterial Adhesion. Mol. Microbiol. 83, 7–9. 10.1111/j.1365-2958.2011.07913.x 22092444

[B62] Kovach M. E. Elzer P. H. Hill D. S. Robertson G. T. Farris M. A. Roop R. M. 2. (1995). Four New Derivatives of the Broad-Host-Range Cloning Vector pBBR1MCS, Carrying Different Antibiotic-Resistance Cassettes. Gene 166, 175–176. 10.1016/0378-1119(95)00584-1 8529885

[B63] Kumar S. Stecher G. Li M. Knyaz C. Tamura K. (2018). Mega X: Molecular Evolutionary Genetics Analysis Across Computing Platforms. Mol. Biol. Evol. 35, 1547–1549. 10.1093/molbev/msy096 29722887 PMC5967553

[B64] Leyton D. L. Rossiter A. E. Henderson I. R. (2012). From Self Sufficiency to Dependence: Mechanisms and Factors Important for Autotransporter Biogenesis. Nat. Rev. Microbiol. 10, 213–225. 10.1038/nrmicro2733 22337167

[B65] Liautard J. P. Gross A. Dornand J. Kohler S. (1996). Interactions Between Professional Phagocytes and Brucella Spp. Microbiologia 12, 197–206. 8767704

[B66] Liu Z. Wei D. Li Y. Zhou H. Huang D. Guan P. (2020). Different Clinical Manifestations of Human Brucellosis in Pregnant Women: A Systematic Scoping Review of 521 Cases From 10 Countries. Infect. Drug Resist. 13, 1067–1079. 10.2147/IDR.S248779 32341659 PMC7166055

[B67] Livak K. J. Schmittgen T. D. (2001). Analysis of Relative Gene Expression Data Using Real-Time Quantitative PCR and the 2(-Delta Delta C(T)) Method. Methods 25, 402–408. 10.1006/meth.2001.1262 11846609

[B68] Lopez P. Guaimas F. Czibener C. Ugalde J. E. (2020). A Genomic Island in Brucella Involved in the Adhesion to Host Cells: Identification of a New Adhesin and a Translocation Factor. Cell Microbiol., 22, e13245. 10.1111/cmi.13245 32657513

[B69] Lulu A. R. Araj G. F. Khateeb M. I. Mustafa M. Y. Yusuf A. R. Fenech F. F. (1988). Human *Brucellosis* in Kuwait: A Prospective Study of 400 Cases. Q. J. Med. 66, 39–54. 10.1093/oxfordjournals.qjmed.a068178 3051080

[B70] Madeira F. Park Y. M. Lee J. Buso N. Gur T. Madhusoodanan N.. (2019). The EMBL-EBI Search and Sequence Analysis Tools APIs in 2019. Nucleic Acids Res. 47, W636–W641. 10.1093/nar/gkz268 30976793 PMC6602479

[B73] Martirosyan A. Moreno E. Gorvel J. P. (2011). An Evolutionary Strategy for a Stealthy Intracellular *Brucella* Pathogen. Immunol. Rev. 240, 211–234. 10.1111/j.1600-065X.2010.00982.x 21349096

[B74] Mccarthy J. E. Brimacombe R. (1994). Prokaryotic Translation: The Interactive Pathway Leading to Initiation. Trends Genet. 10, 402–407. 10.1016/0168-9525(94)90057-4 7809946

[B75] Merhej V. Royer-Carenzi M. Pontarotti P. Raoult D. (2009). Massive Comparative Genomic Analysis Reveals Convergent Evolution of Specialized Bacteria. Biol. Direct 4, 13. 10.1186/1745-6150-4-13 19361336 PMC2688493

[B76] Meuskens I. Saragliadis A. Leo J. C. Linke D. (2019). Type V Secretion Systems: An Overview of Passenger Domain Functions. Front. Microbiol. 10, 1163. 10.3389/fmicb.2019.01163 31214135 PMC6555100

[B77] Moreno E. M. I. (2001). The prokaryotes: An evolving microbiological resource for the microbiological community (New York: Springer).

[B78] Moreno E. Cloeckaert A. Moriyon I. (2002). *Brucella* Evolution and Taxonomy. Vet. Microbiol. 90, 209–227. 10.1016/S0378-1135(02)00210-9 12414145

[B80] O’callaghan D. Whatmore A. M. (2011). Brucella Genomics as We Enter the Multi-Genome Era. Brief Funct. Genomics 10, 334–341. 10.1093/bfgp/elr026 21930657

[B81] O’toole G. A. Pratt L. A. Watnick P. I. Newman D. K. Weaver V. B. Kolter R. (1999). Genetic Approaches to Study of Biofilms. Methods Enzymol. 310, 91–109. 10.1016/S0076-6879(99)10008-9 10547784

[B82] Paixao T. A. Roux C. M. Den Hartigh A. B. Sankaran-Walters S. Dandekar S. Santos R. L.. (2009). Establishment of Systemic *Brucella melitensis* Infection Through the Digestive Tract Requires Urease, the Type IV Secretion System, and Lipopolysaccharide O Antigen. Infect. Immun. 77, 4197–4208. 10.1128/IAI.00417-09 19651862 PMC2747930

[B83] Pappas G. Papadimitriou P. Akritidis N. Christou L. Tsianos E. V. (2006). The New Global Map of Human *Brucellosis* . Lancet Infect. Dis. 6, 91–99. 10.1016/S1473-3099(06)70382-6 16439329

[B84] Patel S. Mathivanan N. Goyal A. (2017). Bacterial Adhesins, the Pathogenic Weapons to Trick Host Defense Arsenal. BioMed. Pharmacother. 93, 763–771. 10.1016/j.biopha.2017.06.102 28709130

[B85] Paulsen I. T. Seshadri R. Nelson K. E. Eisen J. A. Heidelberg J. F. Read T. D.. (2002). The *Brucella suis* Genome Reveals Fundamental Similarities Between Animal and Plant Pathogens and Symbionts. Proc. Natl. Acad. Sci. U.S.A. 99, 13148–13153. 10.1073/pnas.192319099 12271122 PMC130601

[B86] Pizarro-Cerda J. Cossart P. (2006). Bacterial Adhesion and Entry Into Host Cells. Cell 124, 715–727. 10.1016/j.cell.2006.02.012 16497583

[B87] Posadas D. M. Ruiz-Ranwez V. Bonomi H. R. Martin F. A. Zorreguieta A. (2012). BmaC, a Novel Autotransporter of *Brucella suis*, is Involved in Bacterial Adhesion to Host Cells. Cell Microbiol. 14, 965–982. 10.1111/j.1462-5822.2012.01771.x 22321605

[B800] Prentki P. Krisch H. M. (1984). In Vitro Insertional Mutagenesis with a Selectable DNA Fragment. Gene 29, 303–313. 6237955 10.1016/0378-1119(84)90059-3

[B88] Quandt J. Hynes M. F. (1993). Versatile Suicide Vectors Which Allow Direct Selection for Gene Replacement in Gram-Negative Bacteria. Gene 127, 15–21. 10.1016/0378-1119(93)90611-6 8486283

[B89] Rajashekara G. Glasner J. D. Glover D. A. Splitter G. A. (2004). Comparative Whole-Genome Hybridization Reveals Genomic Islands in Brucella Species. J. Bacteriol. 186, 5040–5051. 10.1128/JB.186.15.5040-5051.2004 15262941 PMC451633

[B91] Ruiz-Ranwez V. Posadas D. M. Estein S. M. Abdian P. L. Martin F. A. Zorreguieta A. (2013a). The BtaF Trimeric Autotransporter of *Brucella suis* Is Involved in Attachment to Various Surfaces, Resistance to Serum and Virulence. PloS One 8, e79770. 10.1371/journal.pone.0079770 24236157 PMC3827427

[B92] Ruiz-Ranwez V. Posadas D. M. Van Der Henst C. Estein S. M. Arocena G. M. Abdian P. L.. (2013b). BtaE, an Adhesin That Belongs to the Trimeric Autotransporter Family, is Required for Full Virulence and Defines a Specific Adhesive Pole of *Brucella suis* . Infect. Immun. 81, 996–1007. 10.1128/IAI.01241-12 23319562 PMC3584859

[B93] Saitou N. Nei M. (1987). The Neighbor-Joining Method: A New Method for Reconstructing Phylogenetic Trees. Mol. Biol. Evol. 4, 406–425. 10.1093/oxfordjournals.molbev.a040454 3447015

[B94] Salcedo S. P. Chevrier N. Lacerda T. L. Ben Amara A. Gerart S. Gorvel V. A.. (2013). Pathogenic *Brucellae* Replicate in Human Trophoblasts. J. Infect. Dis. 207, 1075–1083. 10.1093/infdis/jit007 23303808

[B95] Sarram M. Feiz J. Foruzandeh M. Gazanfarpour P. (1974). Intrauterine Fetal Infection With *Brucella melitensis* as a Possible Cause of Second-Trimester Abortion. Am. J. Obstet. Gynecol. 119, 657–660. 10.1016/0002-9378(74)90128-8 4857794

[B96] Scian R. Barrionuevo P. Fossati C. A. Giambartolomei G. H. Delpino M. V. (2012). *Brucella abortus* Invasion of Osteoblasts Inhibits Bone Formation. Infect. Immun. 80, 2333–2345. 10.1128/IAI.00208-12 22547546 PMC3416452

[B97] Scian R. Barrionuevo P. Giambartolomei G. H. De Simone E. A. Vanzulli S. I. Fossati C. A.. (2011). Potential Role of Fibroblast-Like Synoviocytes in Joint Damage Induced by *Brucella abortus* Infection Through Production and Induction of Matrix Metalloproteinases. Infect. Immun. 79, 3619–3632. 10.1128/IAI.05408-11 21730088 PMC3165475

[B98] Scian R. Barrionuevo P. Rodriguez A. M. Arriola Benitez P. C. Garcia Samartino C. Fossati C. A.. (2013). *Brucella abortus* Invasion of Synoviocytes Inhibits Apoptosis and Induces Bone Resorption Through RANKL Expression. Infect. Immun. 81, 1940–1951. 10.1128/IAI.01366-12 23509146 PMC3676039

[B99] Selkrig J. Mosbahi K. Webb C. T. Belousoff M. J. Perry A. J. Wells T. J.. (2012). Discovery of an Archetypal Protein Transport System in Bacterial Outer Membranes. Nat. Struct. Mol. Biol. 19, 506–510, S501. 10.1038/nsmb.2261 22466966

[B100] Serruto D. Spadafina T. Scarselli M. Bambini S. Comanducci M. Hohle S.. (2009). Hada is an Atypical New Multifunctional Trimeric Coiled-Coil Adhesin of *Haemophilus Influenzae* Biogroup Aegyptius, Which Promotes Entry Into Host Cells. Cell Microbiol. 11, 1044–1063. 10.1111/j.1462-5822.2009.01306.x 19290916

[B101] Sieira R. Bialer M. G. Roset M. S. Ruiz-Ranwez V. Langer T. Arocena G. M.. (2017). Combinatorial Control of Adhesion of *Brucella abortus* 2308 to Host Cells by Transcriptional Rewiring of the Trimeric Autotransporter Btae Gene. Mol. Microbiol. 103, 553–565. 10.1111/mmi.13576 27862467

[B102] Skerker J. M. Laub M. T. (2004). Cell-Cycle Progression and the Generation of Asymmetry in *Caulobacter crescentus* . Nat. Rev. Microbiol. 2, 325–337. 10.1038/nrmicro864 15031731

[B103] Steinhauer J. Agha R. Pham T. Varga A. W. Goldberg M. B. (1999). The Unipolar Shigella Surface Protein IcsA is Targeted Directly to the Bacterial Old Pole: IcsP Cleavage of IcsA Occurs Over the Entire Bacterial Surface. Mol. Microbiol. 32, 367–377. 10.1046/j.1365-2958.1999.01356.x 10231492

[B104] Tomlinson A. D. Fuqua C. (2009). Mechanisms and Regulation of Polar Surface Attachment in *Agrobacterium tumefaciens* . Curr. Opin. Microbiol. 12, 708–714. 10.1016/j.mib.2009.09.014 19879182 PMC2783196

[B105] Tsolis R. M. Young G. M. Solnick J. V. Baumler A. J. (2008). From Bench to Bedside: Stealth of Enteroinvasive Pathogens. Nat. Rev. Microbiol. 6, 883–892. 10.1038/nrmicro2012 18955984

[B106] Van Der Henst C. Beaufay F. Mignolet J. Didembourg C. Colinet J. Hallet B.. (2012). The Histidine Kinase PdhS Controls Cell Cycle Progression of the Pathogenic *Alphaproteobacterium Brucella abortus* . J. Bacteriol. 194, 5305–5314. 10.1128/JB.00699-12 22843843 PMC3457221

[B107] Van Der Henst C. De Barsy M. Zorreguieta A. Letesson J. J. De Bolle X. (2013). The *Brucella* Pathogens are Polarized Bacteria. Microbes Infect. 15, 998–1004. 10.1016/j.micinf.2013.10.008 24141086

[B108] Verger J. M. M. G. (1985). *Brucella*, a Monospecific Genus as Shown by Deoxyribonucleic Acid Hybridization. Int. J. Syst. Bacteriol. 35, 292–295. 10.1099/00207713-35-3-292

[B109] Von Bargen K. Gorvel J. P. Salcedo S. P. (2012). Internal Affairs: Investigating the *Brucella* Intracellular Lifestyle. FEMS Microbiol. Rev. 36, 533–562. 10.1111/j.1574-6976.2012.00334.x 22373010

[B110] Whatmore A. M. (2009). Current Understanding of the Genetic Diversity of *Brucella*, an Expanding Genus of Zoonotic Pathogens. Infect. Genet. Evol. 9, 1168–1184. 10.1016/j.meegid.2009.07.001 19628055

[B111] Williams D. L. Slayden R. A. Amin A. Martinez A. N. Pittman T. L. Mira A.. (2009). Implications of High Level Pseudogene Transcription in *Mycobacterium leprae* . BMC Genomics 10, 397. 10.1186/1471-2164-10-397 19706172 PMC2753549

[B112] Wong G. L. Cohn D. V. (1975). Target Cells in Bone for Parathormone and Calcitonin are Different: Enrichment for Each Cell Type by Sequential Digestion of Mouse Calvaria and Selective Adhesion to Polymeric Surfaces. Proc. Natl. Acad. Sci. U. S. A. 72, 3167–3171. 10.1073/pnas.72.8.3167 171656 PMC432942

[B113] Zuckerkandl E. Pauling L. (1965). Molecules as Documents of Evolutionary History. J. Theor. Biol. 8, 357–366. 10.1016/0022-5193(65)90083-4 5876245

