# Supplementary material for: Adhesive Functions or Pseudogenization of Type Va Autotransporters in Brucella Species
Source: Front Cell Infect Microbiol. 2021 Apr 27;11:607610. doi: 10.3389/fcimb.2021.607610 (PMC8111173; doi:10.3389/fcimb.2021.607610)
Supplement: Supplementary file 1 [file Image_1.pdf]

Figure S1

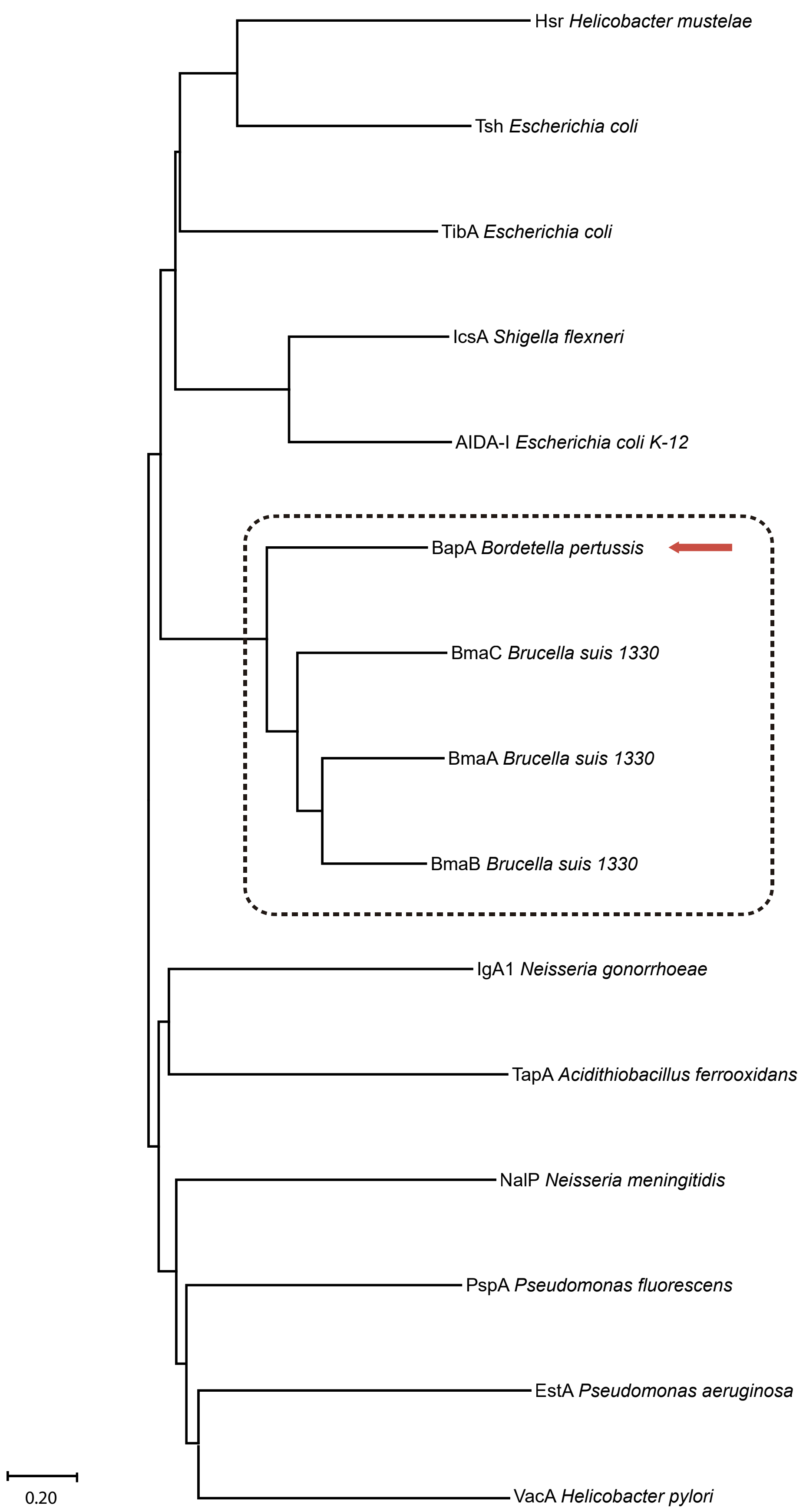

Supplementary figure 1: Evolutionary relationships of beta barrels from type Va autotransporters. Phylogeny was inferred using the Neighbor-Joining method (Saitou & Nei, 1987). The scale bar represents the number of amino acid substitutions per site. The dotted line square highlights the position of the Bma proteins within the tree. The red arrow indicates the recognized autotransporter of the group of the barrel-domain "type" to which the Bma proteins belong (BapA).
