# Supplementary material for: Adhesive Functions or Pseudogenization of Type Va Autotransporters in Brucella Species
Source: Front Cell Infect Microbiol. 2021 Apr 27;11:607610. doi: 10.3389/fcimb.2021.607610 (PMC8111173; doi:10.3389/fcimb.2021.607610)
Supplement: Supplementary file 2 [file Image_2.pdf]

Figure S2

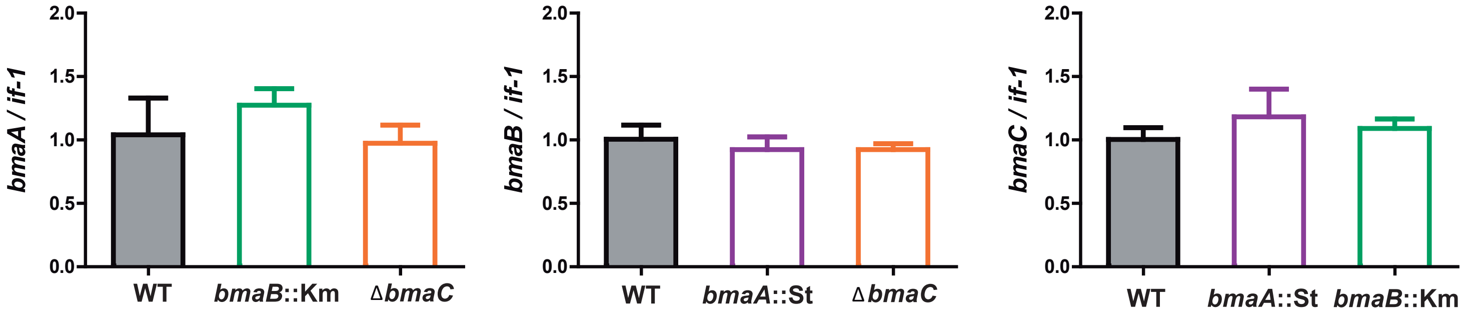

Supplementary Figure 2: **Expression level of *bma* genes in *B.suis* 1330 wt and *bma* mutant strains**.
Mean expression levels of *bmaA*, *bmaB* and *bmaC* genes were measured using quantitative RT-PCR (RT-qPCR) for the wt and the mutant strains. Four biological replicates were used for measuring mRNA abundance.
Each gene measure was normalized to the expression of the *Translation Initiation Factor 1 (If-1)*.
Error bars indicate SEM. Student's *t*-test was performed between mutants and wild type strains.
