# Supplementary material for: Adhesive Functions or Pseudogenization of Type Va Autotransporters in Brucella Species
Source: Front Cell Infect Microbiol. 2021 Apr 27;11:607610. doi: 10.3389/fcimb.2021.607610 (PMC8111173; doi:10.3389/fcimb.2021.607610)
Supplement: Supplementary file 3 [file Image_3.pdf]

Figure S3

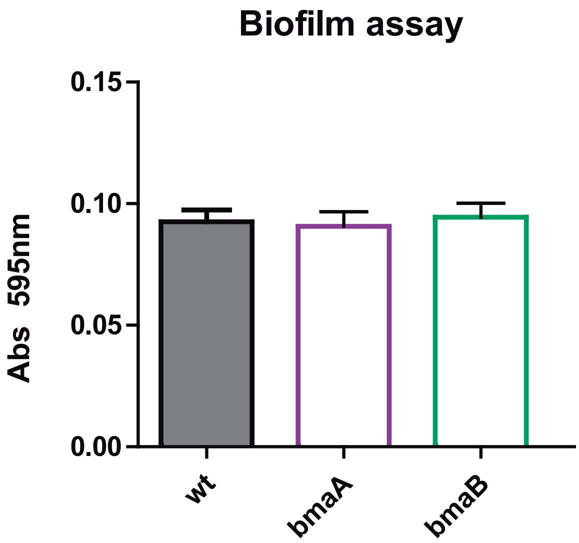

Supplementary Figure 3: **Adherence of *B. suis* strains to an abiotic surface.**
Binding to polystyrene (PE) microtiter plates of *B. suis* wild type, *bmaA*::stops and *bmaB*::Km was assayed.
Attached bacteria were quantified by staining with crystal violet.
Data represent the means and standard deviations (SD) of the results of a representative experiment done in triplicate.
Three independent experiments were performed with similar results.
