# Supplementary material for: Adhesive Functions or Pseudogenization of Type Va Autotransporters in Brucella Species
Source: Front Cell Infect Microbiol. 2021 Apr 27;11:607610. doi: 10.3389/fcimb.2021.607610 (PMC8111173; doi:10.3389/fcimb.2021.607610)
Supplement: Supplementary file 4 [file Image_4.pdf]

Figure S4

A.

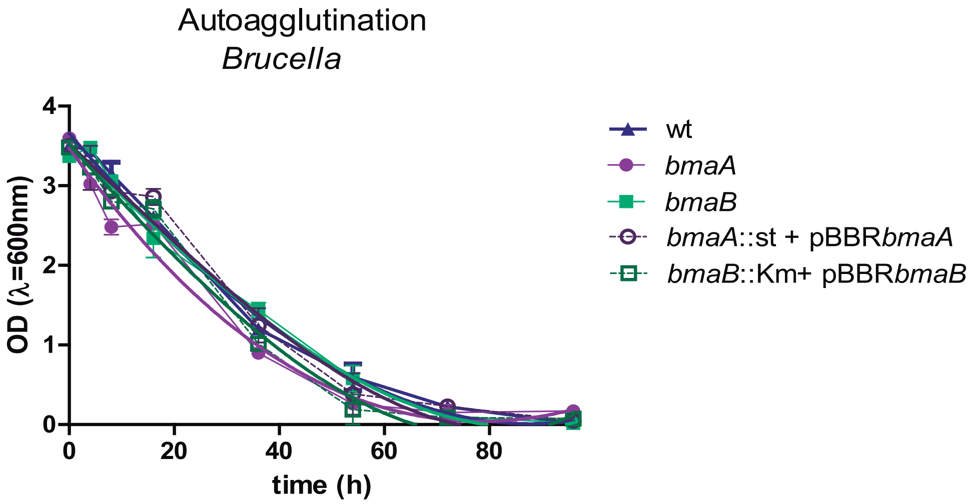

B.

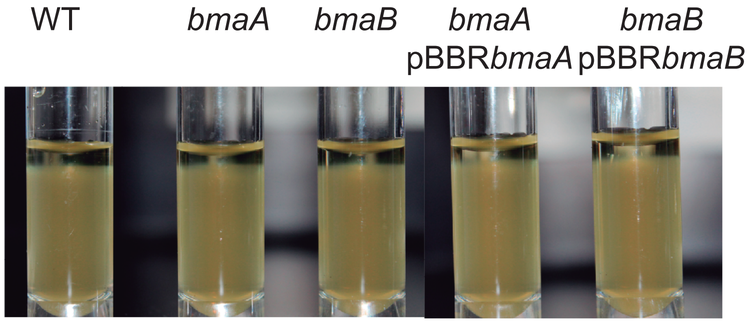

C.

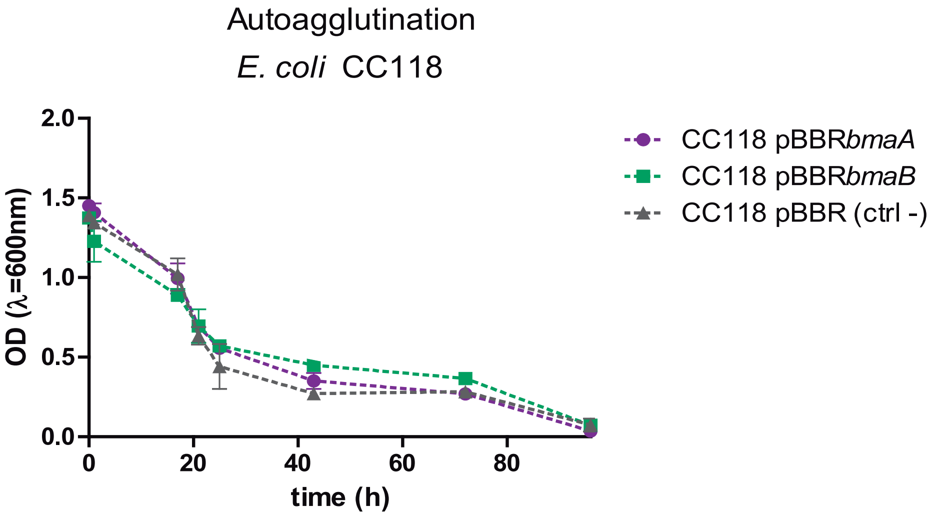

Supplementary Figure 4: **Autoagglutination analysis.**
The sedimentation ability of (A) *B. suis* wt, *bmaA*::stops (*bmaA*) and *bmaB*::Km (*bmaB*) and the complemented mutants; and (C) *E. coli* CC118 pBBR1, *E. coli* CC118 pBBR1*bmaA*, *E. coli* CC118 pBBR1*bmaB* was assayed.
Bacterial sedimentation was measured comparing the optical density (OD600) in the upper portion of the tubes at different time points for a period of 96 hours.
(B) The image corresponds to *Brucella* cultures grown in a rich medium and was let stand for 48 h.
