# Supplementary material for: Adhesive Functions or Pseudogenization of Type Va Autotransporters in Brucella Species
Source: Front Cell Infect Microbiol. 2021 Apr 27;11:607610. doi: 10.3389/fcimb.2021.607610 (PMC8111173; doi:10.3389/fcimb.2021.607610)
Supplement: Supplementary file 5 [file Image_5.pdf]

Figure S5

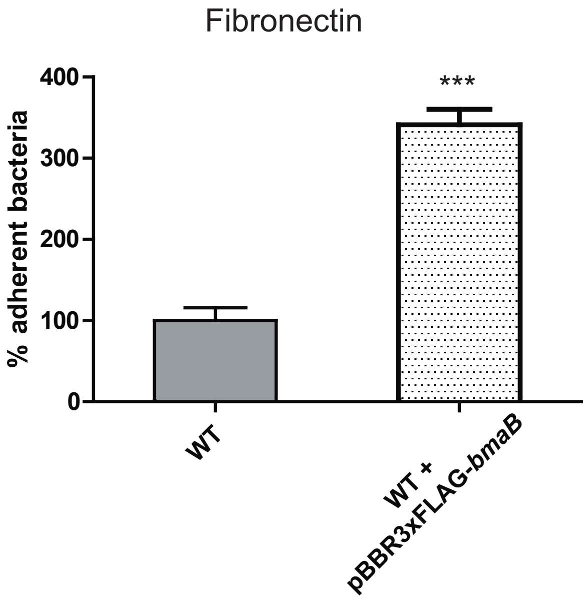

Supplementary Figure 5: **Adherence of *B. suis* wild type (WT) and *B. suis* pBBR3xFLAG-*bmaB* to fibronectin.** Values correspond to the percentage of binding. A value of 100% was assigned to the wild type strain.
Data represent the means and standard deviations (SD) of the results of a representative experiment done in triplicate. Three independent experiments were performed with similar results. Data were analyzed by one-way ANOVA followed by a Tukey's *post hoc* test. ***, significantly different from control ($p < 0.0001$).
