# Supplementary material for: Adhesive Functions or Pseudogenization of Type Va Autotransporters in Brucella Species
Source: Front Cell Infect Microbiol. 2021 Apr 27;11:607610. doi: 10.3389/fcimb.2021.607610 (PMC8111173; doi:10.3389/fcimb.2021.607610)
Supplement: Supplementary file 6 [file Image_6.pdf]

Figure S6

(A) 3xF BmaB localization in *E. coli* CC118

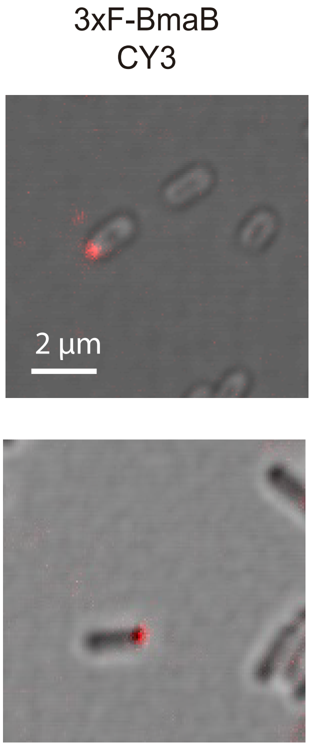

(B)

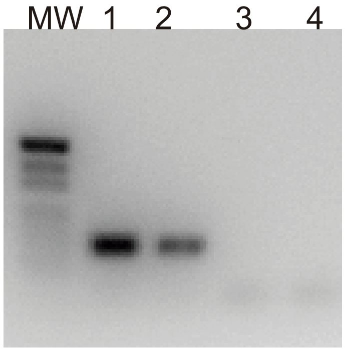

Supplementary Figure 6: Polar localization and transcription analysis of BmaB in heterologous model.

(A) Immunofluorescence assays with anti-FLAG antibodies were performed in *E. coli* CC118 + pBBR3xFLAG-*bmaB* without fixation (upper panel) or after fixation with 4% PFA (lower panel). The shape of the bacteria was observed by DIC images. The cells were observed by confocal microscopy using a ZEISS LSM 880 Confocal Laser Scanning Microscope. (B) Expression of *bmaA* and *bmaB* genes was measured by RT-PCR in *E. coli* CC118 strain expressing (1) pBBR*bmaA*, (2) pBBR*bmaB* and (3 and 4) pBBR1(empty vector).
Oligonucleotides used in 1 and 3 were RT*bmaA* (F and R) and oligonucleotides used in 2 and 4 were RT*bmaB* 4 (F and R) (Table S1).
