# Supplementary material for: Adhesive Functions or Pseudogenization of Type Va Autotransporters in Brucella Species
Source: Front Cell Infect Microbiol. 2021 Apr 27;11:607610. doi: 10.3389/fcimb.2021.607610 (PMC8111173; doi:10.3389/fcimb.2021.607610)
Supplement: Supplementary file 7 [file Image_7.pdf]

Figure S7

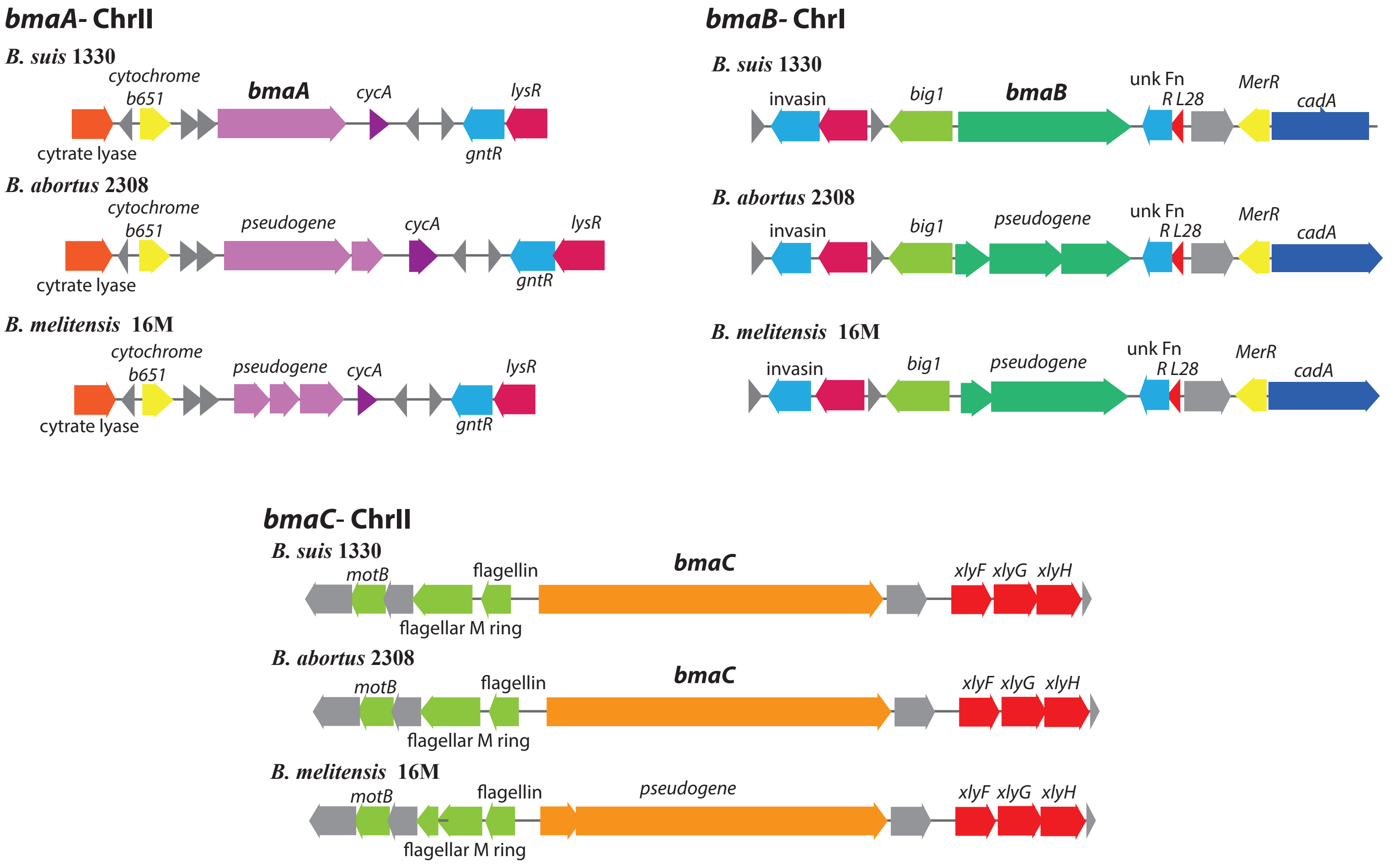

Supplementary Figure 7: **Synteny analysis.**
Relative positions of *bmaA*, *bmaB* and *bmaC* are shown for *B. suis* 1330, *B. abortus* 2308 and *B. melitensis* 16M.
Five genes are shown upstream and downstream of each adhesin.
The name or function of each of the known genes are indicated in the figure. Hypothetical proteins are indicated in gray.
The analysis of the 30 strains used for pseudogene determination revealed that the synteny was conserved in all cases with few variations.
