# Supplementary material for: Adhesive Functions or Pseudogenization of Type Va Autotransporters in Brucella Species
Source: Front Cell Infect Microbiol. 2021 Apr 27;11:607610. doi: 10.3389/fcimb.2021.607610 (PMC8111173; doi:10.3389/fcimb.2021.607610)
Supplement: Supplementary file 8 [file Image_8.pdf]

Figure S8

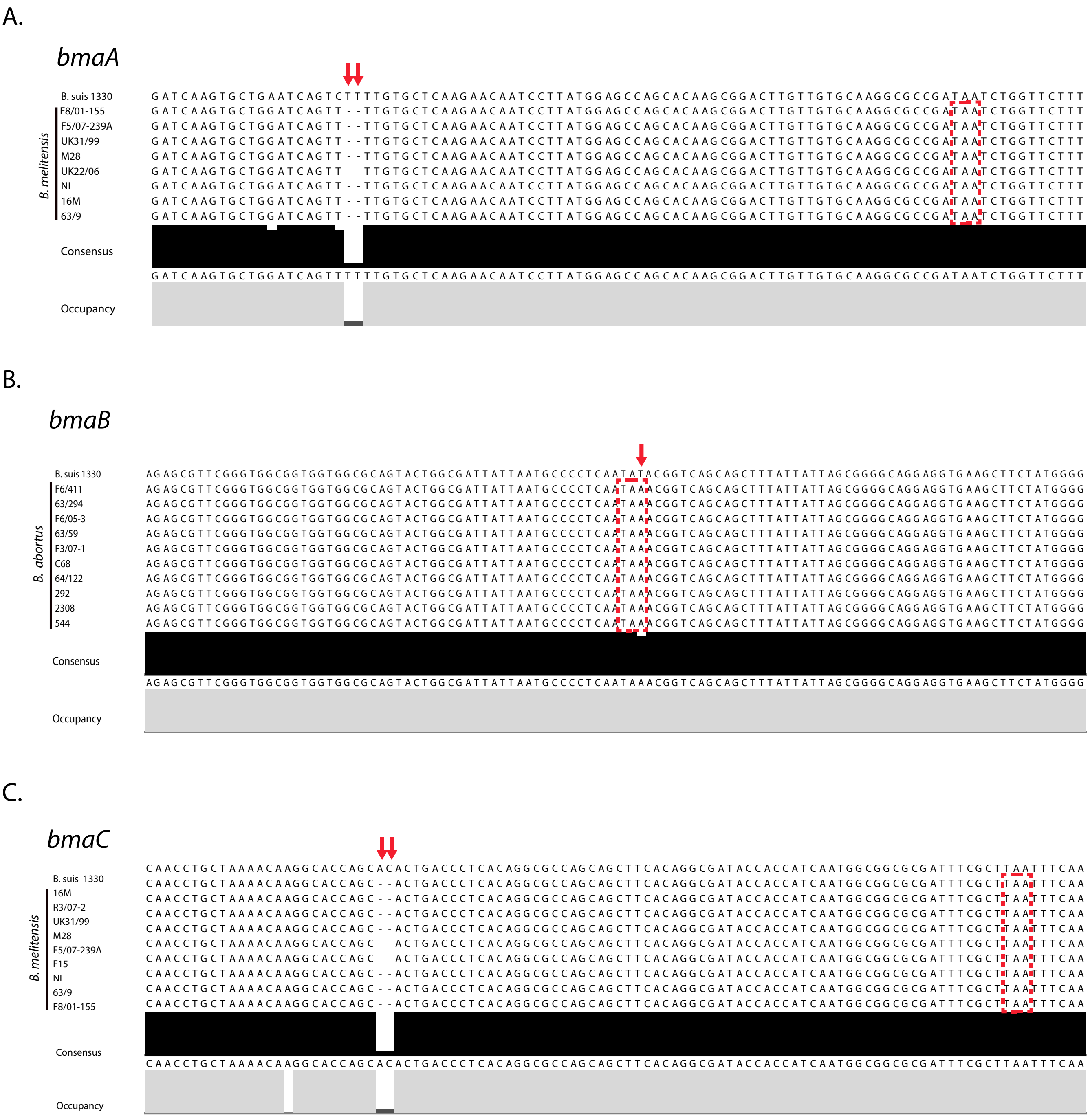

Supplementary figure 8:

Multiple alignments of conserved mutations of (A) *bmaA* orthologues of *B. melitensis*, (B) *bmaB* orthologues of *B. abortus* and (C) *bmaC* orthologues of *B. melitensis*. Conserved mutations are pointed by red arrows and non-sense mutations generated de novo are marked by dotted line squares.
